# Type 2 Diabetes‐Associated Phenylacetylglutamine Induces Deleterious Inflammation Cycle in Myeloid Cells through β_2_ Adrenergic Receptors and Impedes Wound Healing

**DOI:** 10.1002/advs.202508205

**Published:** 2025-08-14

**Authors:** Lu Huang, Xinran Ye, Chia‐Kang Ho, Ya Gao, Dongsheng Wen, Jiaming Sun, Yuxin Liu, Yangdan Liu, Guoyuan Wang, Yangbai Sun, Jinyan Zhang, Yifan Zhang, Qingfeng Li

**Affiliations:** ^1^ Department of Plastic and Reconstructive Surgery Shanghai Ninth People's Hospital Shanghai Jiao Tong University School of Medicine Shanghai China; ^2^ Department of Ophthalmology Schepens Eye Research Institute of Massachusetts Eye and Ear Harvard Medical School Boston MA USA; ^3^ Luo Ping County People's Hospital Qujing Yunnan China; ^4^ Department of Musculoskeletal Surgery Fudan University Shanghai Cancer Center Shanghai China; ^5^ Department of Cardiology Zhongshan Hospital Fudan University Shanghai Institute of Cardiovascular Diseases Shanghai China

**Keywords:** adrenergic receptors, innate immunity training, phenylacetylglutamine, type 2 diabetes, wound healing

## Abstract

Despite advances in glucose‐lowering therapies, many diabetic patients still suffer inflammation‐related complications such as chronic non‐healing wounds. The microbiota‐derived metabolite phenylacetylglutamine (PAGln) is identified as a causal driver of these wounds via a transmissible, β_2_‐adrenergic receptor–mediated trained‐immunity loop. Metabolomics reveals PAGln is elevated in type 2 diabetes and tightly associated with poor healing in both diabetic and non‐diabetic human patients. Pharmacokinetic comparison shows that mouse PAGln exposure closely matches the concentrations and kinetics seen in humans. It is validated that PAGln delays wound closure, impairs collagen restoration, and reduces neovascularization in wild‐type, T1DM, and T2DM mice—defects rescued by β‐blocker treatment. Mechanistically, PAGln epigenetically trains myeloid cells via β_2_‐adrenergic receptors, promoting their hyperresponsiveness, heightening systemic inflammation. This epigenetic reprogramming extends to HSCs, expanding hyper‐responsive myeloid cells and sustaining a myeloid‐biased inflammatory loop. This PAGln‐induced hyper‐inflammatory, wound‐healing deficit is transmissible via bone marrow transplantation to irradiated naïve recipients, confirming that PAGln‐trained hematopoietic cells propagate these deleterious phenotypes. β‐blockers co‐treatment reverses PAGln‐induced cytokine elevation and wound‐healing deficits. These findings link a diet‐derived microbial metabolite, PAGln, to chronic wound and inflammation in T2DM and PAA‐related clinical conditions and highlight β‐blockade as a readily translatable therapy to disrupt the PAGln‐driven deleterious inflammation cycle.

## Graphical abstract

The microbiota‐derived metabolite phenylacetylglutamine (PAGln) is elevated in type 2 diabetes and tightly associated with poor healing in both diabetic and non‐diabetic human patients. PAGln promotes mouse inflammation and impairs healing through a transmissible β_2_‐adrenergic receptor–mediated trained‐immunity loop. β‐blockers disrupt this cycle, offering a translatable therapeutic strategy against metabolite‐induced chronic inflammation.

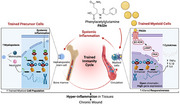

## 1 Introduction

Chronic wounds represent a significant clinical and economic burden, accounting for ≈$19 billion annually, over 3% of healthcare expenditures in industrialized nations.^[^ ^1^ ^]^ Type 2 diabetes mellitus (T2DM) is a major risk factor for chronic wounds, contributing to more than 90% of non‐traumatic lower‐limb amputations.^[^ ^2^ ^]^ Notably, even with optimal glycemic control, many T2DM patients continue to suffer from non‐healing wounds characterized by persistent local hyperinflammation.^[^ ^3^, ^4^, ^5^, ^6^, ^7^ ^]^ These observations suggest the existence of glucose‐independent metabolic disruptions that exacerbate proinflammatory responsiveness and delay wound healing, highlighting an urgent need for a mechanistic dissection of this glucose‐independent hyperinflammation and healing impairment phenotype.

Recent studies have indicated the role of metabolic stressors in exacerbating proinflammatory responsiveness. Metabolites such as lipids, advanced glycation end‐products, and diet‐derived molecules can induce maladaptive “innate immune training”—a state in which innate immune cells undergo long‐lasting epigenetic modifications that enhance proinflammatory gene accessibility, allowing a faster and increased transcriptional proinflammatory responsiveness after rechallenge.^[^ ^8^, ^9^, ^10^ ^]^ Prior findings further suggested that systemic inflammation could epigenetically rewire immune progenitor cells in bone marrow, enhancing myeloid cell production (myelopoiesis) and contributing to immune dysfunction.^[^ ^11^ ^]^ Widespread overnutrition in industrialized societies has amplified the health burden associated with metabolite‐driven hyperinflammation, intensifying the need for mechanistic investigation on how endogenous metabolites exacerbate proinflammatory responsiveness.

Among emerging metabolic candidates, phenylacetylglutamine (PAGln) has gained increasing attention. PAGln came from the conjugation of gut microbiota‐derived or exogenous phenylacetic acid (PAA) to glutamine, and is elevated in individuals with high protein intake^[^ ^12^, ^13^ ^]^ or those receiving PAA‐based nitrogen‐scavenging therapy because of hyperammonemia.^[^ ^14^ ^]^ Elevated circulating PAGln has been associated with a wide range of hyperinflammation‐related health issues, including cardiovascular complications in renal disease,^[^ ^15^ ^]^ colorectal carcinogenesis,^[^ ^16^ ^]^ major adverse cardiac events,^[^ ^17^ ^]^ increased mortality in acute respiratory distress, and diabetes.^[^ ^18^ ^]^ Previous work, particularly the study by Nemet et al., demonstrated that PAGln binds α‐, β₁‐, and β_2_‐adrenergic receptors (ADRs) on platelets to promote thrombosis and increase cardiovascular risk.^[^ ^18^ ^]^ Despite extensive research, whether PAGln can act on ADRs in immune cells, induce immune dysfunction, or play its role in non‐cardiovascular diseases, such as impaired wound healing, has yet to be confirmed.

Here, we identify PAGln, which is elevated in T2DM patients, as a mechanistic link between dietary metabolism and hyperinflammation in the context of chronic wounds in humans and mice (Figure S1, Supporting Information). Mechanistically, PAGln activates β_2_‐ADRs on myeloid cells, epigenetically trains them to be proinflammatory hyperresponsive, resulting in heightened cytokine production and a sustained inflammatory state that extends to epigenetic reprogramming in bone marrow progenitor cells, promoting myelopoiesis, expanding the number of myeloid cells, and thus forming the deleterious cycle. This trained immunity leads to sustained changes that bias increasing myeloid cells toward a pro‐inflammatory state. Upon exposure to wound stimuli, these trained cells exhibit an exaggerated inflammatory response, prolonging the inflammatory phase and hindering the transition to proliferative and remodeling phases.^[^ ^19^ ^]^ The resulting cytokine overload disrupts collagen deposition and tissue repair, ultimately delaying wound healing.^[^ ^19^ ^]^ Bone marrow transplantation confirms the transmissibility of this trained phenotype. Importantly, pharmacological blockade of β_2_‐adrenergic signaling could disrupt this deleterious cycle, suggesting a new therapeutic avenue for reversing hyperinflammation and promoting wound healing. Together, our findings uncover a causal and transmissible link between amino acid metabolism, innate immune training‐related inflammation cycle, and impaired wound healing, extending prior findings on PAGln‐adrenergic signaling to a new pathological context and uncovering a metabolite‐immune axis with therapeutic potential in chronic wounds, providing a new paradigm for understanding and treating T2DM‐ and PAA‐related inflammatory disorders.

## 2 Results

### 2.1 PAGln is Associated with Chronic Wounds in Humans

**Figure 1:**
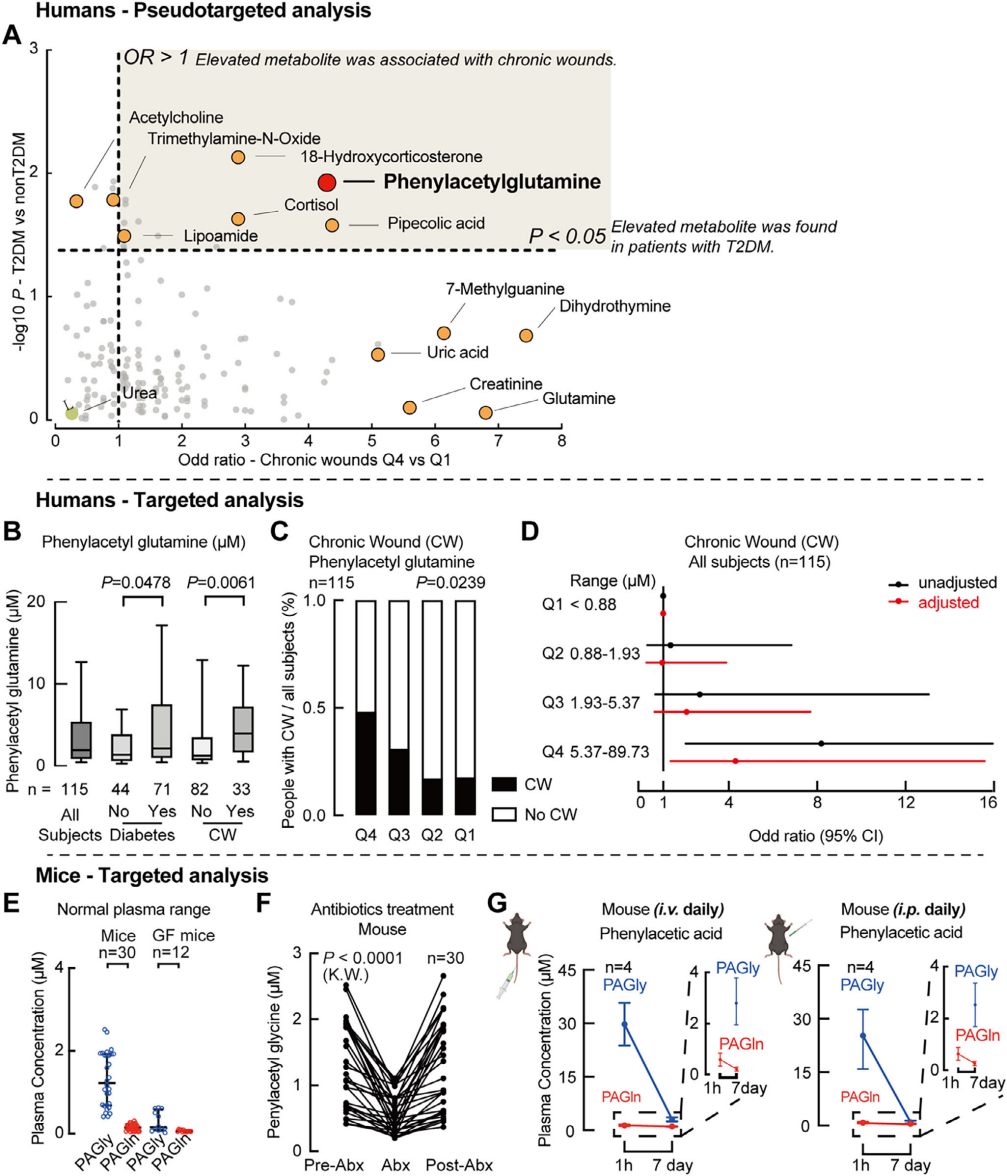
Pseudo‐targeted metabolomics identifies phenylacetylglutamine (PAGln) as a target metabolite associated with chronic wounds. A) Discovery cohort (n = 85): Association between elevated metabolite levels and chronic wounds or type 2 diabetes mellitus (T2DM). The x‐axis shows odds ratios (OR, Q4 versus Q1, 95% confidence interval (95% CI) detailed in Table S2, Supporting Information) for chronic wounds; the y‐axis indicates significance (p‐value) of elevated metabolites in diabetic versus non‐diabetic subjects. B) Relative plasma levels of PAGln in validation subjects (n = 115). Subjects were grouped based on diabetic status or the presence of chronic wounds. Box‐and‐whisker plots indicate medians (horizontal lines), 25th–75th percentiles (box boundaries), and 10th–90th percentiles (whiskers). Statistical significance was assessed by the Mann‐Whitney test. C) Stacked bar plot illustrating chronic wound risk across PAGln quartiles measured by stable isotope dilution LC‐MS/MS (Q4 versus Q1, log‐rank test). D) Logistic regression analysis showing chronic wound risk by PAGln quartiles in validation subjects (n = 115). Unadjusted odds ratios (black); adjusted odds ratios controlling for age, sex, smoking, hypertension, and hyperlipidemia (red). Confidence intervals (CI, 2.5–97.5%) are indicated by line length. E) Fasting plasma concentrations (mean ± s.e.m.) of PAGln and phenylacetylglycine (PAGly) in conventionally raised (n = 30) and germ‐free (GF; n = 12) mice. F) Plasma PAGly levels measured in male (n = 15) and female (n = 15) mice at baseline (Pre‐Abx), after 5‐day broad‐spectrum antibiotic treatment (Abx), and one week post‐antibiotics (Post‐Abx). Statistical significance was assessed by the Kruskal–Wallis test. G) Time course of plasma PAGln and PAGly following intraperitoneal (i.p.) or intravenous (i.v.) injection of phenylacetic acid (50 mg k^−1^g; n = 5 per condition and time point) in mice. The diagram was created in BioRender. https://www.biorender.com/. See also Figure S2 (Supporting Information).

To explore the metabolic signatures linked to chronic wounds, we first performed pseudo‐targeted metabolomics on plasma from a Discovery cohort of 85 individuals undergoing wound status evaluation (Table S1, Supporting Information). Plasma metabolites were prioritized based on two criteria: (1) individuals in the highest quartile (Q4) had a greater chronic wound prevalence than those in the lowest quartile (Q1); and (2) metabolite levels were significantly elevated in patients with T2DM compared to non‐diabetics (Table S2, Supporting Information). Among all analytes, PAGln emerged as a leading candidate, showing an adjusted odds ratio of 4.136 for chronic wound association and elevated in T2DM patients (**Figure** **1**A; Figure S2A–C, Supporting Information).

To validate these findings, we developed a quantitative stable isotope dilution liquid chromatography‐tandem mass spectrometry (LC/MS) assay and applied it to an independent Validation cohort of 115 individuals (Table S3, Supporting Information). Plasma PAGln levels were consistently elevated in individuals with T2DM and those with chronic wounds (Figure 1B), and higher PAGln levels correlated with increased chronic wound prevalence (Figure 1C). Notably, this relationship remained significant after stratification by diabetic status and adjustment for conventional risk factors, indicating that higher PAGln levels might be an independent marker of chronic wounds (Figure 1D; Figure S2D,E and Table S4, Supporting Information). Together, our clinical data established the correlation but not the causation of PAGln to diabetes and chronic wounds.

### 2.2 Dual Pathways of PAGln Biosynthesis in Rodents and Humans

To investigate PAGln biosynthesis, we evaluated gut microbiota‐dependent and ‐independent routes in rodents. In germ‐free and antibiotic‐treated mice, serum levels of phenylacetyl glycine (PAGly)—a rodent analog of PAGln—were nearly abolished, underscoring microbial dependence (Figure 1E,F). In line with species‐specific liver enzyme substrate preference, PAGly was more abundant than PAGln in mouse plasma (Figure 1E), and systemic PAA administration led to PAGly accumulation (Figure 1G). Together with prior reports, our data support that the microbial dependence comes from gut microbe‐dependent PAA, which is derived from dietary phenylalanine (Phe) conversion by gut microbiota, specifically *Clostridium sporogenes*.^[^ ^14^ ^]^ Given that PAA is also used clinically to facilitate urinary nitrogen excretion through glutamine conjugation in humans,^[^ ^13^, ^20^ ^]^ we modeled clinical use in mice and confirmed that exogenous PAA‐derived PAGly formation (Figure 1G). Together with prior studies,^[^ ^13^, ^14^, ^17^, ^20^ ^]^ our results support that PAGln and PAGly are synthesized via conjugation of gut microbe‐derived or exogenous PAA to glutamine in humans and glycine in rodents, respectively (Figures S2F,S3A, Supporting Information).

Because both peak concentration and exposure duration are critical determinants of PAA‐related adverse events ,^[^ ^21^ ^]^ and plasma PAA concentrations in mice are approximately six times higher than PAA levels in the liver and other tissues, ^[^ ^22^ ^]^ we profiled mouse plasma PAA pharmacokinetics following a 50 mg kg^−1^ i.p. dose, consistent with prior studies on PAGly/PAGln‐mediated effects.^[^ ^17^, ^23^ ^]^ Peak plasma PAA reached 88.9 ± 25.8 µM (1210 µg dL^−1^) at 30 min, exceeding the clinical PAA‐related adverse events threshold of 500 µg dL^−1^ (Table S5, Figure S2G, Supporting Information). While PAA in mice primarily yields PAGly rather than PAGln, our goal here is not to treat PAGly as a functional analogue of PAGln but to determine how long‐lasting the systemic consequences of PAA exposure are in vivo, information that is essential for understanding PAA metabolism and its natural history in humans. (Table S6, Figure S2H, Supporting Information). Together, these data provide a pharmacokinetic framework for modelling human PAA exposure risk assessment in PAA‐related therapeutic interventions.

### 2.3 PAGln Drives Hyperinflammation and Delays Wound Healing In Vivo

While our human data support an association between T2DM and elevated PAGln, the diabetic mouse model did not show the same trend, and we did not observe elevated PAGln or PAGly levels in diabetic mouse models (Figure S2I, Supporting Information). This species difference is likely due to the complex interaction between diet, microbiota, and host metabolism. In humans with T2DM, factors such as high‐fat, high‐sugar diets and altered gut microbiome composition are well‐documented and may contribute to elevated PAGln levels.^[^ ^17^ ^]^ To further investigate microbial dependence, we treated diabetic mice with a broad‐spectrum antibiotic cocktail, which significantly reduced plasma PAGly levels but did not accelerate wound closure (Figure S2J, Supporting Information), underscoring microbial dependence also existed in the diabetic mice, but prolonged antibiotic exposure can dampen adaptive immunity^[^ ^25^ ^]^ and fibroblast activity,^[^ ^24^ ^]^ which may mask any benefit from lowered PAGly.

**Figure 2:**
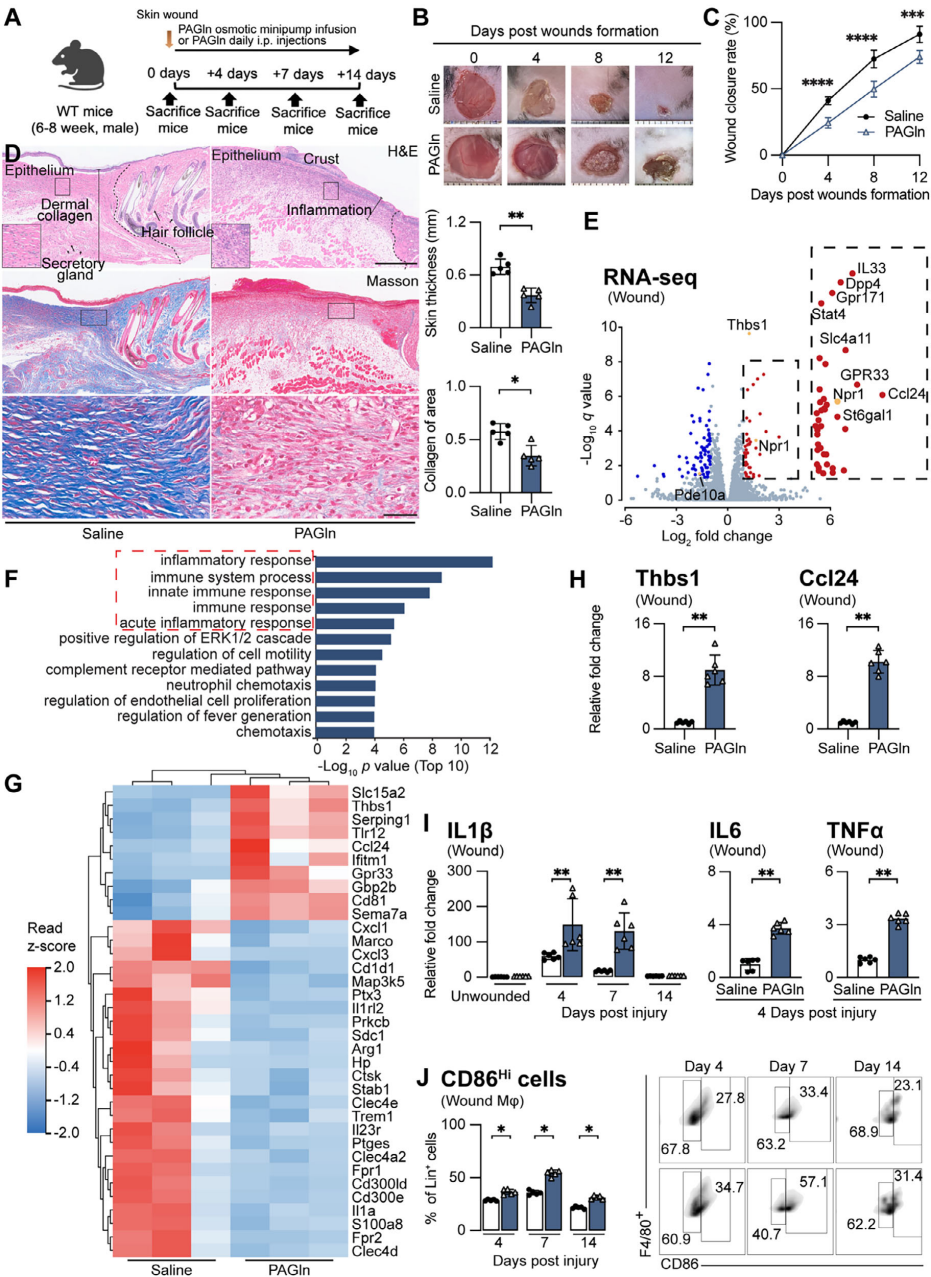
PAGln induces exaggerated proinflammatory responsiveness in dermal wounds. A–D) PAGln impairs wound healing. A) Schematic representation of dermal punch biopsy model. The diagram was created in BioRender. https://www.biorender.com/. B) Representative images of wound closure (n = 6). C) Quantification of wound closure (mean ± s.e.m., n = 5 mice per condition, 3 independent experiments; two‐way ANOVA). D) Representative images (top two scale bar: 500 µm; bottom scale bar: 50 µm) and quantification of skin thickness and dermal collagen (mean ± s.e.m., n = 5 mice per condition, 3 independent experiments). E–H) Gene expression changes induced by PAGln. E) Volcano plot of RNA‐seq data from wound tissues of PAGln‐treated or vehicle‐treated mice on 4 days post‐injury. Genes significantly changed (*p* ≤ 0.05, log2‐fold change ≥ +1 or ≤ ‐1) highlighted in red (upregulated) or blue (downregulated); intracellular cyclic adenosine monophosphate (cAMP)‐related modulators (*Npr1*, *Pde10a*, *Thbs1*) shown in orange. The magnified area is shown in the dotted area. F) Top 10 enriched GO terms among differentially expressed genes. G) Heatmap of DEGs in the top 5 terms in the GO analysis (as dotted). H) Relative mRNA expression of the DEGs found in the top 5 terms in the Gene Ontology (GO) analysis. I) Relative mRNA expression of inflammatory cytokines in wounds at days 4, 7, and 14 after PAGln treatment (mean ± s.e.m., n = 5–6 mice per condition, 3 independent experiments). J) Quantified frequencies (left) and representative flow cytometry plots (right) of CD86^hi^ macrophages percentage among CD45^+^CD11b^+^F4/80^+^ cells in wounds (mean ± s.e.m., n = 5 mice per condition, 3 independent experiments). Gating strategy provided in Figure S4 (Supporting Information). Two‐way ANOVA for wound closure, ***IL1β*** gene expression, and percentage of CD86^hi^ macrophages, Mann–Whitney test for pairwise comparisons; *p < 0.05, **p < 0.01, ***p < 0.001, ****p < 0.0001. See also Figures S3–S6 (Supporting Information).

Because endogenous PAGln is not elevated in mice, we retain the exogenous‐PAGln models to achieve human‐relevant exposure, maintaining plasma levels at ≥ 5.3 µM, the Q4 threshold associated with chronic wounds in humans (Figure 1D). Daily intraperitoneal injection or continuous mini‐pump infusion sustained this concentration throughout the study (Figure S2K,M, Supporting Information). Pharmacokinetic analysis showed that murine PAGln clearance is highly comparable to the human profile reported in a previous study^[^ ^26^ ^]^ (Table S7, Supporting Information). In a dermal punch model, PAGln‐treated mice exhibited delayed wound closure (**Figure** **2**B,C), reduced skin thickness, impaired collagen recovery (Figure 2D), and decreased wound bed neovascularization and perfusion in both diabetic and non‐diabetic models (Figures S3G–O, S9, Supporting Information).

To evaluate specificity, we tested metabolically related or structurally similar metabolites, PAGly, PAA, and Phe, via intraperitoneal injections. Only PAGln, PAGly, and PAA impaired wound healing (Figure S3C,D, Supporting Information). Furthermore, a long‐term exposure mouse model reproduced the same wound‐healing deficits (Figure S3P–R). PAGln infusion did not affect blood glucose or body weight (Figure S3E,F, Supporting Information). Given the similar phenotypes of PAGln and PAGly in mice, we focused on PAGln, as it provides direct insights into the mechanisms of human diseases.

Indeed, RNA sequencing of wound tissue revealed a distinct proinflammatory transcriptomic profile in PAGln‐treated mice (Figure 2E–G), validated by qPCR and flow cytometry (Figure 2H–J; Figure S4, Supporting Information), which revealed upregulated proinflammatory genes and increased proinflammatory macrophage infiltration. No direct effects of PAGln were observed on keratinocyte or fibroblast function (Figure S5, Supporting Information). We indicated that PAGln exacerbates proinflammatory responsiveness, thus drives hyperinflammation and delays wound healing in mice.

### 2.4 PAGln Trains Myeloid Cells to be Proinflammatory Hyperresponsive Through β_2_‐Adrenergic Receptor Signaling

**Figure 3:**
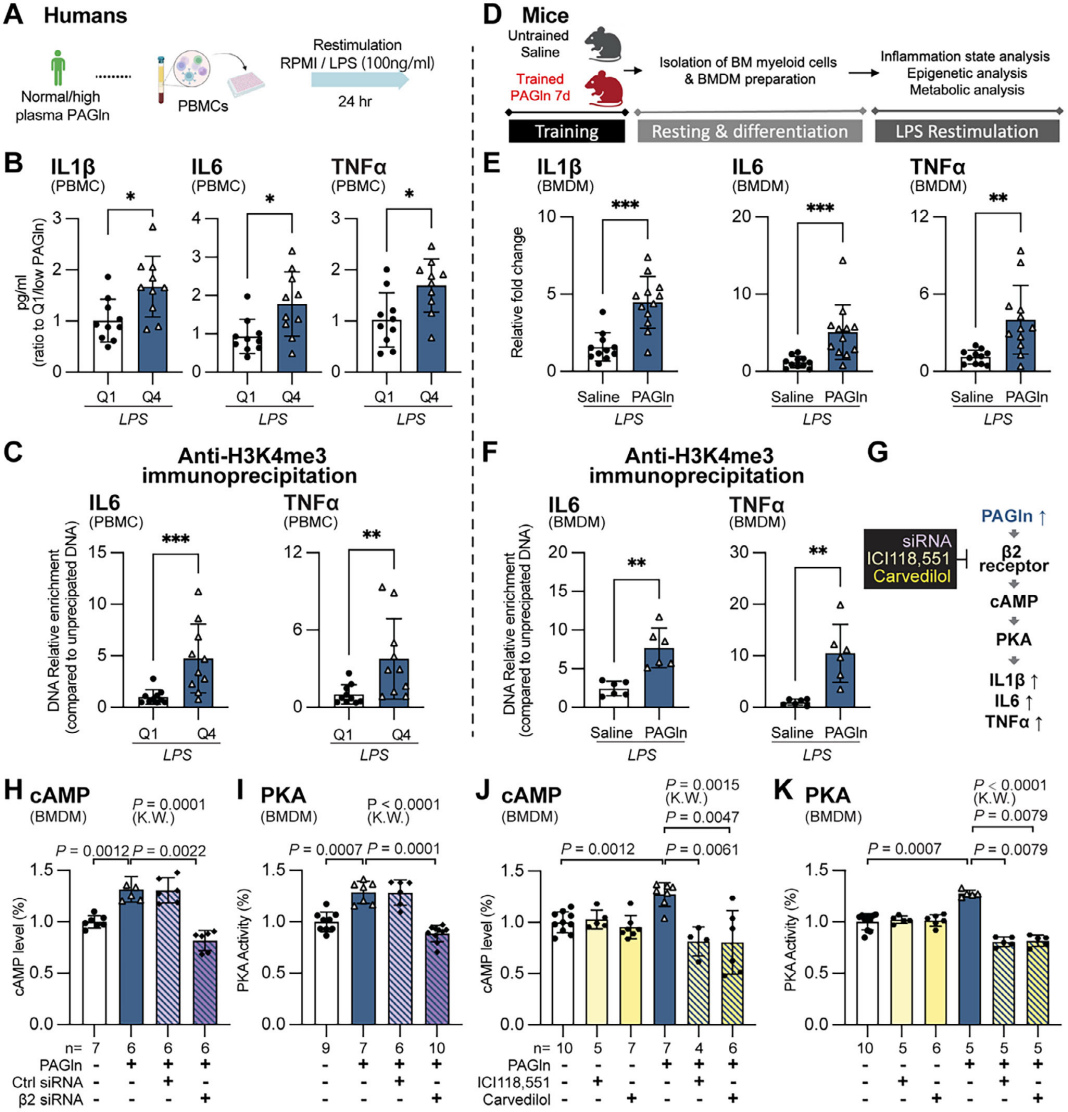
PAGln induces trained immunity via β_2_‐adrenergic receptors in vivo. A–C) Experimental scheme: Human peripheral blood mononuclear cells (PBMCs) from subjects with high (Q4) or low (Q1) plasma PAGln levels restimulated ex vivo with lipopolysaccharide (LPS, 100 ng mL^−1^) for 24 h. B) Cytokine secretion (TNF, IL‐1β, IL‐10; mean ± s.e.m., n = 10 subjects per group). C) H3K4me3 enrichment at IL6 and TNF promoters (mean ± s.e.m., n = 10). D–F) Experimental scheme: Mouse bone marrow‐derived macrophages (BMDM) were isolated and differentiated with M‐CSF for 14 days, then restimulated ex vivo with LPS (100 ng mL^−1^, 6 h). E) Cytokine mRNA expression in PAGln‐trained versus saline‐treated mice (mean ± s.e.m., n = 10 mice per group). F) H3K4me3 enrichment at IL6 and TNF promoters (mean ± s.e.m., n = 6 mice). G) Model illustrating β_2_‐adrenergic receptor‐mediated induction of trained immunity by PAGln through cAMP‐Protein Kinase A (PKA) signaling pathway. H–K) cAMP levels H,J) and PKA activity I,K) in BMDMs transfected with control or β_2_‐adrenergic receptor‐targeted siRNA H,I), or treated with PAGln (100 µm, 5 min) in the presence of selective β_2_ antagonist ICI118551 (10 µM) or nonselective β‐blocker carvedilol (10 µM) J,K). Data represent mean ± s.e.m., n = 4–10 mice per condition, 3 independent experiments. Mann–Whitney test for pairwise comparisons, Kruskal–Wallis test for multiple comparisons; *p < 0.05, **p < 0.01, ***p < 0.001, ****p < 0.0001. The diagram in panel A, D was created in BioRender. https://www.biorender.com/. See also Figure S7 (Supporting Information).

We then investigated whether myeloid cells previously exposed to PAGln could elicit enhanced responses to subsequent challenges. In humans, monocytes from individuals in the highest PAGln quartile secreted significantly more cytokines upon LPS stimulation than those from the lowest quartile, suggesting PAGln‐related innate immune memory (**Figure** **3**A,B; Figure S10D,E, Supporting Information). Monocytes from individuals with chronic wounds secreted significantly more cytokines upon LPS stimulation than those without chronic wounds, suggesting chronic wound‐associated innate immune memory (Figure S10D,E, Supporting Information). The PAGln‐related, chronic wounds‐associated myeloid cell hyperresponsive still existed in T2DM patients (Figure S10F,G, Supporting Information). PAGln exposure enhanced H3K4me3 marks at proinflammatory genes, indicating epigenetic rewiring (Figure 3C). In mice, bone marrow‐derived macrophages (BMDMs) from PAGln‐pretreated animals displayed elevated cytokine expression and H3K4me3 enrichment on proinflammatory genes following LPS rechallenge (Figure 3D–F). These results indicate that elevated PAGln not only marks but also potentiates the hyper‐inflammatory state characteristic of chronic non‐healing wounds in T2DM,.We showed that PAGln rewires the epigenetic modifications of myeloid cells in both mouse and human, training them to an exaggerated inflammatory response upon rechallenge.

PAGln has previously been shown to directly bind and activate β_2_‐ADR in HEK293T cells and platelets;^[^ ^17^ ^]^ here, we further examine for the first time whether PAGln also activates β_2_‐ADR signaling in myeloid cells. We prioritized β_2_‐ADR for three connected reasons. First, PAGln shares structural similarity with catecholamines (Figure S6I, Supporting Information) and can directly bind adrenergic receptor,^[^ ^17^ ^]^ making β_2_‐ADR a plausible “first‐contact” sensor on wound‐site myeloid cells. Second, β_2_‐ADR is a well‐established upstream regulator of inflammatory signaling,^[^ ^27^ ^]^ and its sustained activation promotes myeloid‐cell training.^[^ ^28^ ^]^ Third, targeting this receptor‐proximal axis allows us to (i) test causality at the initiation step, (ii) obtain a pharmacological handle that modulates rather than broadly suppresses immunity, and (iii) trace how upstream β_2_‐ADR–cAMP–PKA signaling in myeloid cells leads to downstream inflammatory responses at the skin wound level.

Consistent with this, our bulk RNA‐seq and KEGG analysis of skin tissue primarily reflect downstream inflammation‐related pathways, rather than upstream cAMP or Ras signaling, which are specific to β_2_‐ADR activation in myeloid cells (Figure S6J, Supporting Information).

We proved that siRNA‐mediated knockdown of β_2_‐ADRs reduced PAGln‐induced cAMP production and PKA activation in BMDMs (Figure 3H,I; Figure S6N,O, Supporting Information). Pharmacologic β_2_ signaling inhibition using ICI118551 or carvedilol yielded similar results (Figure 3J,K; Figures S6K–M,P, Supporting Information). Additional blockade with cholera toxin (CTX) and SCH‐202676 confirmed Gαs‐mediated GPCR activation (Figure S6C–H, Supporting Information). These effects were recapitulated in human THP‐1 cells and further validated by mimicking the action of PAGln on the ADR‐cAMP‐PKA pathway with ADR agonist isoproterenol (Figure S6, Supporting Information). Combined, using genetic and pharmacologic approaches, we confirmed that PAGln acts on myeloid cells via β_2_‐ADR‐cAMP‐PKA in human and mouse.

To demonstrate that PAGln promotes inflammation in macrophages via β_2_‐ADR, we showed that PAGln increased IL‐1β, TNF‐α, and IL‐6 expression and secretion in BMDMs, whereas β_2_‐ADR knockdown eliminated this effect (Figure S7B,C, Supporting Information). As β_2_‐ADR is a known upstream regulator of the PKA–NF‐κB axis,^[^ ^27^ ^]^ we further found that PAGln activated NF‐κB, which was suppressed by β_2_‐ADR silencing (Figure S7D, Supporting Information). These findings provide direct evidence that PAGln drives proinflammatory cytokine production and signaling through β_2_‐ADR, supporting the KEGG enrichment results (Figure S6J, Supporting Information) and strengthening the mechanistic link between PAGln and impaired wound healing.

Together, these findings establish a direct causal link between PAGln exposure‐induced β_2_ receptor activation, myeloid cell training, proinflammatory hyperresponsiveness, and hyperinflammation. Our findings further suggest that β_2_ blockers might mitigate PAGln‐induced hyperinflammation, underscoring the profound impact of PAGln on immune cell responsiveness and highlighting a potential therapeutic target for it.

### 2.5 PAGln Triggers Myelopoiesis via Epigenetic Reprogramming of HSCs

**Figure 4:**
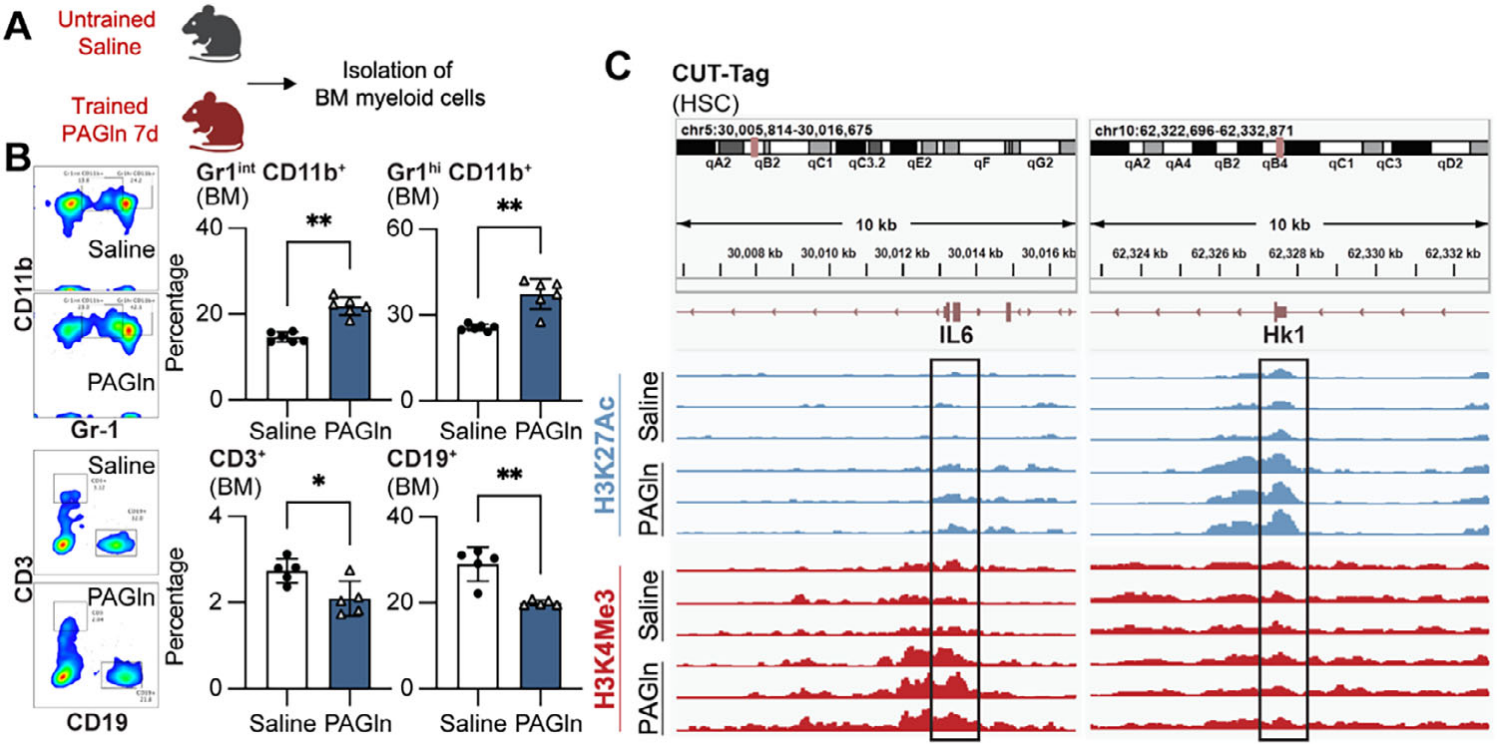
PAGln enhances myelopoiesis and induces epigenetic rewiring of mouse hematopoietic stem cells in vivo. A) Experimental scheme: mice received PAGln or saline for 7 days, followed by bone marrow (BM) analysis. The diagram was created in BioRender. https://www.biorender.com/. B) Representative flow cytometry plots (left) and quantified frequencies (right) of granulocytes (Gr1^hi^CD11b^+^), myeloid cells (Gr1^int^CD11b^+^), B cells (CD19^+^), and T cells (CD3^+^) within CD45^+^ BM cells (mean ± s.e.m., n = 5–6 mice/group, Mann–Whitney test; *p < 0.05, **p < 0.01). C) CUT‐Tag analysis of H3K4me3 and H3K27ac marks at *Il‐6* and *Hk1* loci in hematopoietic stem cells (HSCs). Regions ±0.6 kb around transcription start sites are highlighted (n = 3 mice per group).

Given the short lifespan of mature myeloid cells, the persistence of proinflammatory myeloid cells following PAGln exposure (Figure 2J) suggested upstream reprogramming at the progenitor level within bone marrow (BM). In PAGln‐treated mice, BM progenitor cells showed enhanced myelopoiesis, demonstrated by a higher proportion of Gr1^hi^CD11b^+^ granulocytes and Gr1^int^CD11b^+^ myeloid cells (**Figure** **4**B). We have demonstrated that PAGln modulates myeloid cells via β_2_‐ADRs, triggering systemic inflammation in mice. Historical data suggested that systemic inflammation in mice can epigenetically reprogram HSCs and lead to sustained enhancement of the production of myeloid cells.^[^ ^11^ ^]^ Cut&Tag profiling of HSCs revealed increased H3K4me3 and H3K27ac enrichment at *interleukin‐6 (IL‐6)* and *hexokinase 1 (Hk1)*, key inflammatory and metabolic signaling relevant to trained immunity^[^ ^29^ ^]^ (Figure 4C). These results indicate that PAGln induces epigenetic reprogramming of HSCs, promoting increased circulating myeloid cells that can be further trained by PAGln, thus initiating a deleterious inflammation cycle.

### 2.6 PAGln‐Induced Hyperinflammation and Delayed Healing are Transmissible via Bone Marrow Transplantation

**Figure 5:**
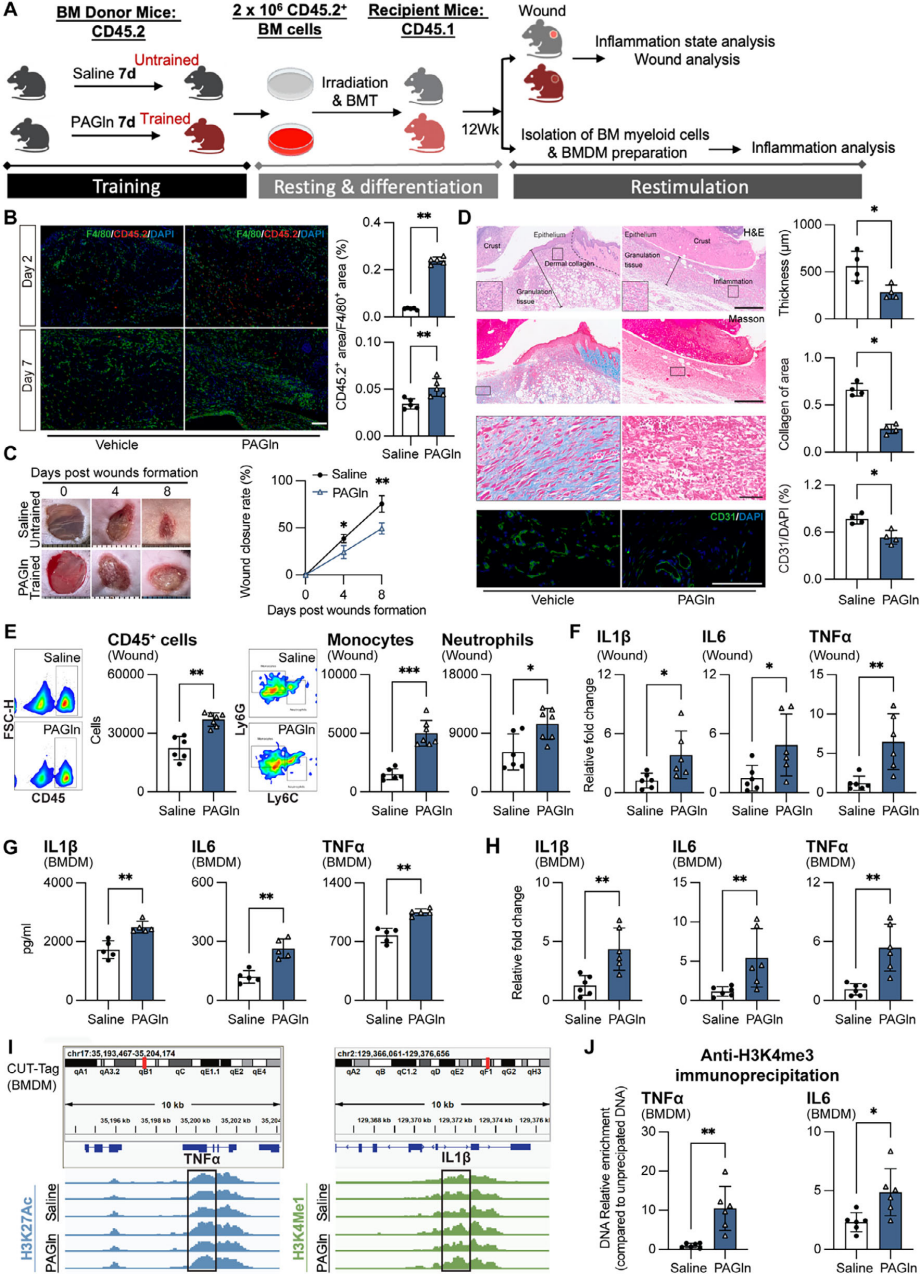
PAGln‐induced impaired wound healing and trained immunity is transmissible through bone marrow transplantation. A) Experimental scheme: CD45.2^+^ donor mice were treated with PAGln or saline for 7 days, and bone marrow (BM) was transplanted into lethally irradiated congenic CD45.1^+^ recipient mice. At 12 weeks post‐BMT, recipient mice underwent cutaneous wound induction, with wound healing parameters assessed 8 days post‐wounding. The diagram was created in BioRender. https://www.biorender.com/. B) Immunofluorescence staining and quantification for CD45.2 and F4/80 in wound tissues. C) Wound closure rate, D) skin thickness, dermal collagen, and vascularization (scale bars: top two images, 500 µm; bottom, 50 and 20 µm), E) flow cytometric analysis of monocytes (live CD45^+^CD11c^−^CD11b^+^Ly6G^−^Ly6C^+^), neutrophils (live CD45^+^CD11c^−^CD11b^+^Ly6G^+^Ly6C^−^), and total CD45^+^ cells, and F) relative mRNA expression of inflammatory cytokines in wound tissues (mean ± s.e.m., n = 6–7 mice per group). G,H) Cytokine G) secretion (IL‐1β, TNF, IL‐6) and H) relative mRNA expression by LPS‐restimulated bone marrow‐derived macrophages (BMDMs) isolated from recipient mice at 12 weeks post‐transplantation (mean ± s.e.m., n = 5–6 mice per group). I) CUT‐Tag analysis of H3K27ac and H3K4me1 marks at *TNF* and *IL‐1β* loci in BMDMs isolated from recipient mice. Regions ±0.6 kb around the transcription start site of *TNF* and *IL‐1β* eRNA locus are highlighted (n = 3 mice per group). F) H3K4me3 enrichment at *TNF* and *IL6* promoters (mean ± s.e.m., n = 6 mice). Two‐way ANOVA for wound closure, Mann–Whitney test for pairwise comparisons; *p < 0.05, **p < 0.01, ***p < 0.001, ****p < 0.0001. See also Figure S8 (Supporting Information).

To test whether PAGln‐trained BM progenitor cells could indeed proceed this deleterious inflammation cycle, we transplanted BM from PAGln‐treated CD45.2^+^ donors into lethally irradiated CD45.1^+^ recipients (**Figure** **5**A). The successful transplantation of BM was validated by immunofluorescence staining with donor marker CD45.2 and F4/80 (Figure 5B). Twelve weeks post‐transplant, recipient mice were subjected to dermal punch biopsy and displayed impaired wound healing, increased immune infiltration, and elevated proinflammatory cytokine expression (Figure 5C–F). BMDMs isolated from these recipients showed heightened inflammatory gene expression, cytokine secretion, and enhanced H3K4me3 marks at proinflammatory genes upon LPS challenge (Figure 5G–J), confirming transmissible PAGln‐induced epigenetic rewiring, hyperinflammation, and delayed healing via BM transplantation.

Together, consistent with prior findings that systemic inflammation can reprogram BM progenitor cells and induce myelopoiesis,^[^ ^11^ ^]^ we demonstrate that PAGln‐induced systemic inflammation reprograms HSCs at the epigenetic level, predisposing them to myeloid bias upon secondary challenge.

### 2.7 Inhibition of β_2_‐Adrenergic Signaling Mitigates PAGln‐Induced Wound Healing Impairment, Hyperinflammation, And Trained Myelopoiesis

**Figure 6:**
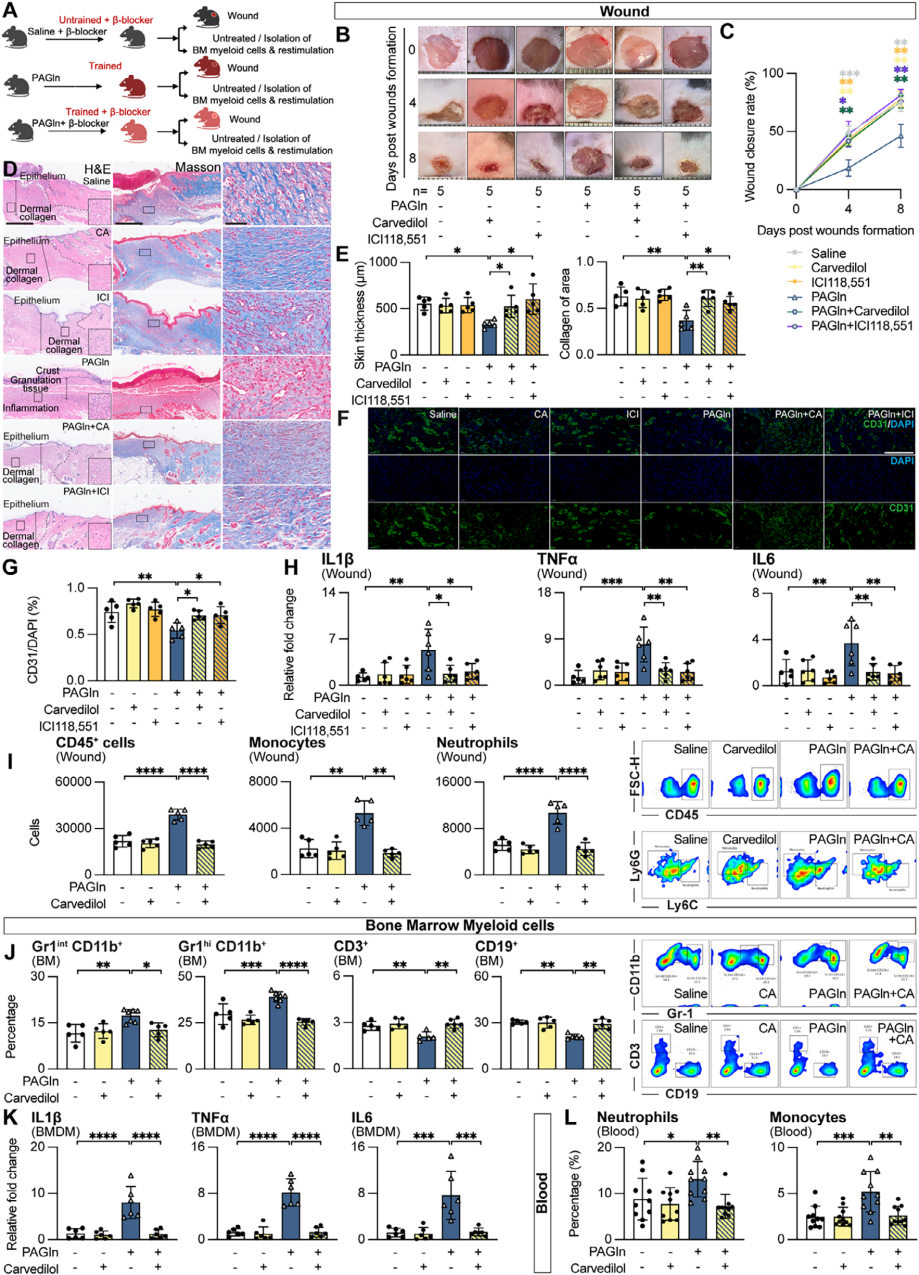
β_2_‐adrenergic receptor inhibition attenuates PAGln‐induced impairment in wound healing, hyperinflammation, and myelopoiesis. A) Experimental scheme: Saline control or PAGln‐trained mice were treated with β_2_‐adrenergic receptor antagonist (carvedilol, 50 mg kg^−1^ day^−1^; ICI118551, 10 mg kg^−1^ day^−1^, intraperitoneal injection for 8 days). The diagram was created in BioRender. https://www.biorender.com/. B–G) β_2_‐adrenergic receptor blockade ameliorates PAGln‐induced impairment in wound healing. B,C) wound closure rate, D–E) skin thickness and dermal collagen (scale bars: 500 µm top images; 50 µm bottom images), F,G) revascularization (scale bar: 20 µm) assessed at day 8 post‐injury (mean ± s.e.m., n = 5 mice per condition, 3 independent experiments). H,I) β_2_‐adrenergic receptor blockade reduces PAGln‐induced hyperinflammation. H) Cytokine mRNA levels and I) flow cytometric analysis of wound monocytes (live CD45^+^CD11c^−^CD11b^+^Ly6G^−^Ly6C^+^), neutrophils (live CD45^+^CD11c^−^CD11b^+^Ly6G^+^Ly6C^−^), and total CD45^+^ cells (mean ± s.e.m., n = 5 mice per condition, 3 independent experiments). J–L) β_2_‐adrenergic receptor blockade suppresses PAGln‐induced myelopoiesis and myeloid cell hyperresponsiveness. J) Myeloid cell frequencies in bone marrow and K) cytokine expression in bone marrow‐derived macrophages (BMDMs) following LPS stimulation (100 ng mL^−1^, 6 h, mean ± s.e.m., n = 5–7 mice per group, 3 independent experiments). L) Blood monocytes and neutrophils percentage among leukocytes (mean ± s.e.m., n = 10 mice per condition, 3 independent experiments). Two‐way ANOVA for wound closure, Mann–Whitney test for pairwise comparisons, Kruskal–Wallis test for multiple comparisons; *p < 0.05, **p < 0.01, ***p < 0.001, ****p < 0.0001. See also Figure S8 (Supporting Information).

Finally, we evaluated the therapeutic potential of β_2_‐ADR inhibitors on PAGln‐induced deleterious inflammation phenotypes. Treatment with either carvedilol or ICI118551 improved wound closure, restored skin thickness, enhanced collagen recovery and neovascularization, and reduced inflammatory cytokine expression and immune infiltration (**Figure** **6**A–I). β_2_‐adrenergic blockade also normalized progenitor cell myelopoiesis (Figure 6J), dampened myeloid cell hyperresponsiveness to LPS (Figure 6K), and restored blood monocyte and neutrophil levels (Figure 6L). These findings highlight β_2_‐adrenergic signaling as a therapeutic target for PAGln‐associated impaired wound healing, hyperinflammation, and myelopoiesis.

## 3 Discussion

The prevalence of metabolic disorders has risen significantly over the past century, yet the mechanisms linking them to chronic inflammation remain largely unclear. Through a multi‐faceted approach, this study combined metabolomics, RNA‐sequence, in vitro studies, animal models, and human samples and identified a gut microbiota‐derived metabolite, PAGln, as a key instigator of this link through adrenergic receptor‐mediated trained immunity. Previous work has shown that PAGln amplifies cardiovascular risk through direct binding to α‐, β₁‐, and β_2_‐ADRs on platelets,^[^ ^17^ ^]^ while trained immunity has been implicated in linking periodontitis and arthritis inflammatory comorbidity.^[^ ^11^ ^]^ Our study builds on this foundation in five principal ways. First, we link circulating PAGln concentrations to hyperinflammation and chronic wounds in both diabetic and non‐diabetic human patients through metabolomics coupled with in‐depth functional myeloid analysis, thereby broadening the clinical and pathological relevance of PAGln beyond the vasculature. Second, we provided direct in vivo experimental evidence establishing the link of PAGln to the exacerbation of inflammation and, for the first time, extended its effect to the impairment of wound repair. Additional inclusion of a long‐term PAGln administration model more accurately reflects chronic, low‐level exposure observed in humans, enhancing the translational relevance of the findings. Third, we provide a comprehensive characterization of the mechanistic work linking PAGln to β_2_‐ADR and subsequently to a deleterious inflammation cycle that extends from mature myeloid cells to HSCs. The bone marrow transplant experiment convincingly demonstrates that the PAGln‐induced changes in stem cells can be functionally transplanted, providing definitive support for the hematopoietic stem cell mechanism, strengthening the central hypothesis. Fourth, we enhance translational fidelity by combining human–mouse PAGln pharmacokinetic comparison with long‐term PAGln delivery, thereby modelling the chronic, low‐level PAGln burden typical of metabolic disease. This study also conducted a deeper exploration of the detailed PAA‐exposure profiling in mice, providing implications for understanding the risk for PAA‐related therapy in humans. Finally, we demonstrate that concurrent β_2_‐adrenergic blockade normalizes PAGln‐induced myelopoiesis, hyper‐inflammation, and rescues wound healing repairment, showing that β‐blockers can counteract PAGln‐driven trained immunity and impaired healing, opening the way for potential therapy for inflammatory complications in T2DM and PAA‐related clinical conditions. Together, these advances deepen the mechanistic, translational, and therapeutic understanding of PAGln while complementing and extending prior research. (Graphic abstract).

PAGln exhibits specificity in the β_2_ ADR–trained immunity‐deleterious inflammation cycle pathway. PAGln operates through a distinct β_2_‐adrenergic route that differs fundamentally from other β–ADR–linked metabolites. For example, tyramine is known to indirectly activate β‐adrenergic receptors by promoting norepinephrine release, then provoking local gut inflammation.^[^ ^30^ ^]^ In contrast, PAGln directly binds β_2_‐ADR as a biased negative allosteric modulator, initiating epigenetic reprogramming at the level of both myeloid cells (through β_2_‐ADR activation) and HSCs (through systemic inflammation). This leads to sustained systemic myeloid‐biased trained immunity, and a transmissible hyperinflammatory phenotype and wound‐healing deficit shown by bone‐marrow‐transfer data. Our findings, therefore, highlight the mechanistic specificity of PAGln among β–ADR–linked metabolites and delineate PAGln as the unique gut‐microbial‐derived metabolite that both allosterically modulates β_2_AR and reprograms HSCs, distinguishing it from indirect β‐ADR activators and highlighting a unique microbiome‐adrenergic‐immunity axis amenable to β‐blocker intervention.

Analyzing clinical data with pseudo‐targeted and targeted metabolomics in two independent populations revealed that elevated plasma PAGln is a robust and independent marker of chronic wounds in both diabetic and non‐diabetic individuals. During the discovery stage, to increase the likelihood of identifying novel metabolic pathways involved in impaired healing, we focused on individuals with diabetes, a vulnerable population particularly prone to delayed or non‐healing wounds. This strategy was guided by the recognition that impaired wound repair in T2DM may be influenced by factors beyond traditional risk factors and glucose levels.^[^ ^31^ ^]^ Mechanistically, PAGln exposure promotes H3K4me3 enrichment at inflammatory gene loci in monocytes and BMDMs, resulting in a hyperresponsive state upon secondary challenge. These findings support the growing evidence that dietary patterns can influence hematopoietic activity, shaping disease progression and inflammatory outcomes,^[^ ^32^, ^33^ ^]^ confirming a causal link between metabolite exposure and sustained immune remodeling.

We discovered that this effect is mediated through β_2_‐ADR signaling and, notably, significantly mitigated by treating animals with the β_2_‐agonists Carvedilol or ICI118551, suggesting β_2_‐ADR as a novel therapeutic target for managing inflammation in T2DM. Previous studies have shown that β_2_‐ADR antagonism can enhance keratinocyte migration, promote re‐epithelialization, and accelerate tissue repair independent of microbial metabolites, both ex vivo in excised human skin and in vivo in murine amputated tail wounds, models that differ from the murine dorsal cutaneous wounds used in our study.^[^ ^34^, ^35^ ^]^ To disentangle the general impact of β_2_‐ADR inhibition itself, we specifically included a control group to show that in our wound healing model, β_2_‐ADR inhibition by itself has no significant effect in murine dorsal cutaneous wounds (Figure 6). While β‐blockers show promise in targeting PAGln‐driven inflammation, systemic use can cause off‐target effects. These include bradycardia, hypotension, and worsened heart failure; bronchospasm in asthma/COPD; fatigue, mood changes, and sleep disturbances; masked hypoglycemia in diabetics; and sexual dysfunction. Abrupt withdrawal may trigger rebound cardiovascular events.^[^ ^36^, ^37^, ^38^ ^]^ Future strategies should explore selective or localized β_2_‐blockade to minimize systemic risks.

We uncover dual biosynthetic pathways for PAGln that demonstrate its broad implications. Endogenously, gut microbes metabolize dietary Phe to PAA, which is then conjugated with glutamine in the liver and generates PAGln in humans,^[^ ^23^ ^]^ seen clinically in conditions such as high‐protein diet‐induced metabolic disorders,^[^ ^14^ ^]^ gut‐liver axis disruption,^[^ ^39^ ^]^ severe constipation,^[^ ^40^ ^]^ patients undergoing sleeve bariatric surgery,^[^ ^41^ ^]^ and intestinal tumors.^[^ ^42^ ^]^ Exogenously, PAA is used clinically in hepatic diseases,^[^ ^43^ ^]^ and urea‐cycle disorders,^[^ ^44^ ^]^ to facilitate nitrogen clearance, resulting in elevated systemic PAGln. PAGln also significantly accumulates in dialysis patients, as renal clearance is impaired.^[^ ^45^ ^]^ We showed that in vivo, PAA, PAGln, and its rodent analog PAGly all impair wound healing, validating their metabolic, structural, and functional relevance. Therefore, the mechanism we uncovered may explain the inflammation observed in response to treatments that artificially elevate PAGln levels. Our findings support the broader concept that dietary components and microbiota‐derived metabolites serve as systemic immune modulators.^[^ ^11^ ^]^ Given PAGln's established causal links to cardiovascular diseases,^[^ ^17^ ^]^ our study expands its pathological relevance to impaired tissue repair. Rather than targeting upstream microbial pathways—a theoretical challenge in “drugging the microbiome”—mitigating downstream PAGln effects through β_2_‐agonists may offer a more practical approach to correcting this metabolite‐induced immune imbalance. We validated in our models that β‐blockers, particularly carvedilol, effectively mitigate PAGln‐induced immune dysfunction and healing impairment, aligning with prior evidence of their benefit in chronic wound care^[^ ^46^ ^]^ and immune modulation.^[^ ^47^ ^]^ These results position β_2_‐adrenergic signaling as a critical mediator of metabolite‐induced immune training.

Several limitations in human data should be acknowledged. First, the diagnosis of chronic wounds in our study was based on clinical criteria without molecular confirmation. Second, the cross‐sectional design of the human study precludes assessment of dynamic changes in metabolite levels over time. Additionally, key patient‐specific factors that could influence metabolomic profiles, such as diet, genetic background, lifestyle, and microbiota composition, were not available and could not be controlled. To minimize variability from meals and circadian rhythms, fasting blood samples were collected between 7:00–10:00 a.m. after ≥10 h of fasting, and all inpatients (n = 87) received standardized meals. Under these controlled conditions, PAGln remained elevated in T2DM and rose in inpatients with chronic wounds (Figure S10A, Supporting Information). Chronic‐wound prevalence increased across PAGln quartiles (Figure S10B, Supporting Information), and multivariate analysis confirmed PAGln as an independent risk factor (Figure S10C, Supporting Information), underscoring the robustness of this association beyond dietary or diurnal influences. Third, while exogenous PAGln administration enabled precise control over plasma levels and achieved the levels comparable to those observed in high‐risk human populations (Q4 ≥ 5.3 µm), this does not fully replicate the complex, chronic exposures observed in humans. Moreover, endogenous PAGln is not elevated in *db/db* diabetic mice, likely due to interspecies differences in diet, microbiota, and liver metabolism. Our preliminary attempts using a high‐fat/high‐sugar diet‐induced diabetic model to study endogenous PAGln elevation yielded inconsistent results (data not shown), likely due to the requirement for both specific gut microbiota and adequate dietary phenylalanine—an essential amino acid necessary for PAGln biosynthesis.^[^ ^17^ ^]^ Given these challenges and our main goal to investigate the mechanistic role of PAGln in wound pathology, we chose to administer PAGln exogenously to achieve controlled and physiologically relevant levels. Fourth, our study primarily aimed to identify plasma risk factors and assess their causal roles in chronic wound pathology, and thus did not examine the direct molecular interactions between β_2_AR–cAMP–PKA signaling and epigenetic regulation. Prior studies have suggested that this pathway may influence epigenetic states by modulating the activity or expression of histone‐modifying enzymes or their transcriptional regulation.^[^ ^48^, ^49^, ^50^, ^51^, ^52^ ^]^ Further investigation into the direct molecular mechanisms underlying this signaling axis may shed light on how metabolic cues or dysregulated metabolites contribute to trained immunity, and potentially inform future therapeutic strategies for increasingly prevalent metabolic disorder‐related inflammatory conditions. Fifth, while our study focused on wound healing as a representative inflammatory condition, the generalizability of the PAGln–β_2_‐ADR–trained immunity axis to other chronic inflammatory diseases remains to be determined. Future studies incorporating high‐fat/high‐sugar/high‐protein diets and microbiota modulation (e.g., defined colonization or fecal transplantation from T2DM patients with high PAGln) are warranted to better mimic human PAGln dynamics. In addition, broader disease contexts should be explored, and longitudinal and interventional human studies with multiple sampling time points are needed to elucidate the factors regulating PAGln levels, define optimal therapeutic windows, and refine β‐blocker–based strategies.

In summary, we reveal a novel mechanistic link between the diet‐derived microbial metabolite PAGln and a deleterious inflammatory cycle mediated by β_2_‐adrenergic receptor activation and innate immune training. These findings identify β_2_‐adrenergic signaling as a therapeutic target for T2DM‐ and PAA‐related inflammation and highlight the potential of β‐blockers to restore immune balance by modulating metabolite–immune circuits. In summary, we reveal a novel direct mechanistic link between diet‐derived microbial metabolite PAGln and a deleterious inflammation cycle mediated by β_2_‐ADR activation and innate immune training, driving chronic inflammation and impaired tissue repair. These findings identify β_2_‐adrenergic signaling as a therapeutic target for T2DM‐ and PAA‐related inflammation, highlighting the potential of β‐blockers to restore innate immune balance by modulating metabolite‐immune circuits.

## 4 Experimental Section

### Human Subjects

Samples of venous blood were drawn between 7:00–10:00 a.m. after an overnight fast of ≥10 h, after informed consent was obtained. The study was approved by the institutional ethics committee (fn. 42 117.006) and complied with the Declaration of Helsinki. PBMCs were isolated by density centrifugation of Ficoll‐Paque (Cytiva) according to standard protocols. The mononuclear cell‐enriched fraction was taken forward for monocyte isolation with a negative selection bead isolation kit (EasySep human monocyte enrichment kit without CD16 depletion, StemCell Technologies, Canada) as described before,^[^ ^30^ ^]^ following the manufacturer's described protocol.

During all in vitro culture assays, human isolated monocytes were kept in RPMI 1640 culture medium supplemented with 50 µg mL^−1^ gentamycin, 2 mm glutamax (Gibco), and 1 mm pyruvate (Gibco) at 37 °C in a 5%CO_2_ atmosphere. Then 5 × 10^5^ PBMCs in a 100‐µL volume were added to round‐bottom 96‐well plates (Greiner) and incubated with 100 µL of stimulus. After 24 h, the supernatants were collected and stored at −20 °C until assayed. PBMCs were either lysed in RLT (QIAGEN), snap‐frozen, and stored at −80 °C until assayed or fixed in 1% methanol‐free formaldehyde and stored in PBS before sonication, after which chromatin was stored at −80 °C.

### Untargeted LC–MS/MS Analysis of Human Plasma Samples

Pseudo‐targeted analysis of plasma samples was described previously.^[^ ^53^ ^]^ Ion‐pair reversed‐phase liquid chromatography (IPRP‐LC) system coupled with a tandem mass spectrometry (MS/MS) detector was used. Plasma samples were processed by deproteinization followed by metabolite extraction, using a 1:1 ratio of methanol to water as the extraction solvent. The extracts were dried under vacuum and reconstituted in a 10% aqueous methanol solution before analysis. Chromatographic separation was performed using an ACQUITY BEH C18 column (100 × 2.1 mm, 1.7 µm) at 50 °C with a flow rate of 0.5 mL min^−1^. The mobile phase consisted of 5 mm hexylamine in water (adjusted to pH 10.2) as solvent A and 10 mm ammonium acetate in methanol (90:10 v/v) as solvent B. The gradient elution was programmed as follows: 0–2 min, 0% B; 2–5 min, 0–20% B; 5–10 min, 20–50% B; 10–12 min, 50–100% B; followed by 2 min re‐equilibration at 0% B. The mass spectrometer was operated in negative ion mode with electrospray ionization. Key parameters included an ion spray voltage of −3500 V, curtain gas at 35 psi, and a capillary temperature of 350 °C. Metabolites were analyzed in the scheduled multiple‐reaction monitoring (sMRM) mode, with retention times predicted based on a quantitative structure‐retention relationship (QSRR) model. Data were acquired and processed using Analyst software, and quantification was performed for 164 metabolites based on linear calibration curves established with quality control samples.

### Targeted LC‐MS/MS Analysis of Human Plasma Samples

Stable‐isotope‐dilution LC‐MS/MS quantified PAGln in human plasma as before.^[^ ^17^ ^]^ Plasma (20 µL) was combined with 80 µL of ice‐cold methanol with D5‐phenylacetylglutamine, vortexed, and centrifuged at 21000 × *g* for 15 min at 4 °C. The supernatant was transferred to micro‐insert glass vials for analysis. Chromatography was conducted on an ACQUITY BEH C18 column, using a mobile phase gradient of 0.1% acetic acid in water and acetonitrile. The gradient was adjusted over 15 min with a flow rate of 0.4 mL min^−1^ and a 1 µL injection volume. Metabolites were monitored using scheduled multiple‐reaction monitoring (sMRM), and data were processed via Analyst software.

### In Vitro Training Experiments

Monocytes were adjusted to 1 × 10^6^ cells mL^−1^. A 100 µL volume was added to flat‐bottom 96‐well plates, and cells were incubated at 37 °C. After 1 h, cells were washed once with 200 µL warm PBS to remove non‐adherent cells. Subsequently, monocytes were incubated with culture medium only (negative control) or 100 ng mL^−1^ Escherichia coli LPS (serotype 055:B5, Sigma‐Aldrich) for 24 h. Subsequently, supernatants were collected and stored at −20 °C until cytokine concentrations were assessed.

### Animals Used

All animal procedures were performed following protocols approved by the Institutional Animal Care and Use Committee (IACUC) of the Shanghai Jiao Tong University School of Medicine. 8‐10‐week‐old Wildtype C57BL/6 J, db/db (BKS‐Lepr^em2Cd479^/Gpt), and C57BL/6JGpt CD45.1^+^ mice were purchased from GemPharmatech (https://en.gempharmatech.com/index.html). Both sexes of animals were used in the experiments. Germ‐free C57BL/6 male mice (8‐10 weeks old) were raised at the GemPharmatech gnotobiotic animal facilities, shipped germ‐free to the laboratory, and sacrificed immediately to get the blood. T1DM was induced through an intraperitoneal injection of streptozotocin at a low dose range (42–45 mg kg^−1^ d^−1^) over 5 consecutive days. After 6 weeks following the final streptozotocin injection, diabetic mice with nonfasted blood glucose levels >13.9 mmol L^−1^ were used for wound healing experiments. The plasma‐equivalent glucose was measured from tail vein blood samples (≈3 µl) of mice using the Clarity GL2PLUS glucose meter. Administer carvedilol (50 mg kg^−1^) or ICI118551 (10 mg kg^−1^) via intraperitoneal injection once a day for a total of 5 consecutive days. A 12‐h light‐dark cycle with free access to food and water was maintained for the animals.

### Cell preparations and Sample Collection

For BM single‐cell suspension or BMDM preparation, femoral and tibial bones of C57BL/6 mice or CD45.1^+^ recipient mice were flushed with ice‐cold PBS (Gibco) supplemented with 5% FBS (Gibco). Cells were forced through a 70‐µm cell strainer (BD PharMingen) to get a single‐cell suspension for further flow cytometric analysis. For culture, BM cells were then resuspended at 10^6^ per ml in complete DMEM (DMEM, SVF 10%, P/S, 1% L‐Glu) supplemented with 10 ng ml^−1^ M‐CSF for 7 days to get BMDMs for in vitro experiments. For HSCs isolation, MagniSort Mouse Hematopoietic Lineage Depletion Kit (ThermoFisher Scientific, UK) was used after flushing BM from murine femurs and tibias and incubating with markers to remove lineage‐committed cells (CD3, CD4, B220, Ter119, and Gr1) and enrich for HSCs. For skin single‐cell suspension, the skin was cleaned of fat tissue and cut into small pieces, incubated in Liberase DH (0.5 mg ml^−1^, Sigma) for 90 min before finally shredding through 70‐µm cell strainers (BD PharMingen). For mouse dermal fibroblasts (MDFs) preparation, embryos were harvested on embryonic day 13.5–14.5 as described before.^[^ ^54^ ^]^ After the heads and most of the internal organs were removed, the remaining tissues were put into 0.25% trypsin‐EDTA overnight at 4 °C and were digested for 30 min at 37 °C the next day. Cells were cultured in Dulbecco's Modified Eagle Medium (DMEM) (Gibco, USA) supplemented with 10% fetal bovine serum (FBS) (Gibco, USA) and 1% penicillin/streptomycin antibiotics (Gibco, USA).

BM‐derived cell lines, THP‐1, human peripheral blood monocytes, HaCaT human keratinocyte cells, and MDFs were cultured in DMEM media and kept in a 5% CO_2_ incubator at 37 °C. Unless otherwise stated, all culture media were supplemented with fetal bovine serum (FBS; 10%), penicillin (100 U mL^−1^), and streptomycin (100 µg mL^−1^).

### Murine Antibiotic Challenge

C57BL/6J male (n = 15), C57BL/6J female (n = 15), and db/db diabetic mice (n = 5) aged 8–10 weeks, received an antibiotic cocktail to inhibit gut microbial growth as detailed before.^[^ ^17^ ^]^ Initially, baseline blood samples were drawn from the saphenous vein into heparin‐treated tubes while mice were on a standard chow diet. Subsequently, the mice consumed drinking water mixed with an antibiotic cocktail—comprising kanamycin (0.4 mg mL^−1^), gentamycin (0.035 mg mL^−1^), colistin (0.057 mg mL^−1^), metronidazole (0.215 mg mL^−1^), vancomycin (0.045 mg mL^−1^), and erythromycin (0.01 mg mL^−1^)—for 5 days. For wound healing assessment, wounds were created on day 5 following antibiotic treatment, which was maintained throughout the wound assessment period. For metabolite measurements, a second blood sample was collected post‐treatment. After discontinuing the antibiotics, the mice returned to normal water. To promote microbial recolonization, fecal pellets from untreated, conventionally raised animals were introduced into their bedding. A third blood sample was collected seven days following antibiotic cessation.

### Murine Wound Healing

Mice were anesthetized, dorsum shaved, cleaned with 70% alcohol, and two 0.8cm‐diameter full‐thickness incisional wounds were made through the skin and left to heal.

### Histological Analysis

Harvested wounds were fixed in 4% buffered formaldehyde. The skins were subsequently paraffin‐embedded and sliced for hematoxylin and eosin (H&E) or Masson's trichrome stain (Masson). Skin thickness was determined by measuring the distance between the epidermal surface and the dermal‐subcutaneous tissue demarcation, using a line perpendicular to these boundaries. Collagen content was assessed by identifying and quantifying blue‐stained collagen fibers within the dermis. For immunofluorescence staining, sections were incubated with a primary antibody against CD31 (Invitrogen, MA5‐37858, 1:200), F4/80 (Invitrogen, MA5‐16363, 1:200), CD45.2 (Invitrogen, 14‐0454‐82, 1:200) overnight at 4 °C, and on the next day, sections were incubated with secondary antibody (donkey anti‐rabbit [A488], A21206, Invitrogen; goat anti‐mouse [A555], A21422, Invitrogen). Sections were finally DAPI mounted (glycerol mounting medium with DAPI, Abcam, UK). Fluorescent images were analyzed with ImageJ software.

### Bulk RNA Sequencing

Two wound tissues from one mouse were pooled, three mice were subjected to PAGln, and 3 untrained saline control mice were used. The skin was cleaned of muscle and fat tissue; total RNA was extracted using TRIzol (Invitrogen, Carlsbad, CA, USA) and purified using a RNeasy Mini Kit (Qiagen, Valencia, CA, USA). PolyA‐selected mRNA libraries were generated following the manufacturer's protocols (BGI). Samples were sequenced on the BGISEQ platform to generate 100 bp paired‐end reads with an average depth of 45.0 m reads per sample. Clean reads were mapped to the mouse genome (Ensembl assembly GRCm38) using Spliced Transcripts Alignment to a Reference with default settings after filtering low‐quality, adaptor‐polluted, and high content of unknown base (N) reads. The average mapping ratio with reference genome was 96.46%, and the average mapping ratio with gene was 80.83%. A total of 16181 genes were detected. Most transcripts were completely covered, and reads were evenly distributed throughout the transcript.

### Flow Cytometry and Sorting

Flow cytometric analysis was performed on a FACS CantoTM flow cytometer (BD Biosciences). For skin inflammatory macrophage analysis, a lineage (Lin) cocktail, including the following monoclonal antibodies, was used: CD45 (30‐F11), CD11b (clone M1/70), and F4/80 (BM8). Other antibody reagents used in experiments included anti‐CD45.1 (clone A20), anti‐CD45.2 (clone 104), anti‐CD3 (clone 17A2), anti‐CD19 (clone 6D5), anti‐CD11b (clone M1/70), anti‐Gr1 (clone RB6‐8C5), anti‐CD11c (clone N418), anti‐Ly6C (clone HK1.4), anti‐Ly6G (clone 1A8), anti‐CD45 (30‐F11), anti‐CD86 (clone GL1) were used. Apoptotic cells were detected using Annexin V/propidium iodide (PI) (BD Biosciences). Data were analyzed with FlowJo software. Gating strategies for neutrophils and monocytes in mouse skin were as follows: neutrophils, live CD45+CD11c−CD11b+Ly6G+Ly6C−; monocytes, live CD45+CD11c−CD11b+Ly6G−Ly6C+.

### mRNA Extraction and RT‐PCR

For restimulation of PBMCs with LPS, 2 × 10^6^ PBMCs were added to flat‐bottom 24‐well plates, and cells were allowed to adhere for 1 h at 37 °C. Subsequently, PBMCs were restimulated with culture medium as a negative control or LPS (100 ng mL^−1^) for 24 h. For in vitro re‐stimulation of BMDMs with LPS, isolated and differentiated BMDMs were seeded into 24‐well plates and stimulated with 100 ng ml^−1^ LPS (InvivoGen) for 6 h.

Total cellular RNA was isolated from mouse tissues and cells using Trizol (Invitrogen) according to the manufacturer's instructions. RNA was reverse‐transcribed using the High‐Capacity RNA‐to‐cDNA Kit (Applied Biosystems), and relative expression of cDNA was measured following the TaqMan Gene Expression Assays protocol with the Applied Biosystems 7500 Fast Real‐Time PCR System. Data were analyzed using the comparative (ΔΔCt) method and were normalized to β‐actin mRNA.

### Laser Doppler Perfusion Imaging (LDPI) and Microblood Flow Scan

Mice were anesthetized with 1–2% isoflurane in oxygen. MoorFLPI‐2 blood flow imager (Moor Instruments, UK) was used to scan the surface of the wound for blood flow. The distance between the scan head and the wound surface was ≈20 cm. The image acquisition rate was one frame per second in the normal resolution mode. The duration of the recording was 1 min.

### In Vitro Assessment of Specific Histone Marks

After restimulation as before, 1 × 10^7^ PBMCs or BMDMs in 10 mL medium were harvested and fixed in 1% methanol‐free formaldehyde. Fixed cell preparations were sonicated using a Diagenode Bioruptor Pico sonicator using five cycles of 30 s on and 30 seconds off, and ChIP was performed using antibodies against H3K4me3 (CST, 9751). A PCR purification Kit (Vazyme, TH904‐C1) was used for DNA isolation. Afterward, qPCR analysis was performed using the SYBR Green method, and samples were analyzed by a comparative Ct method.

### In Vivo Assessment of Genome‐Wide Epigenetic Profiles

For genome‐wide chromatin assessment, the histone 3 lysine 1 trimethylation (H3K4me1), histone 3 lysine 4 trimethylation (H3K4me3), and histone 3 lysine 27 acetylation (H3K27ac) marks were chosed, which were generally associated with an “active” chromatin state at both gene promoters and distal (enhancer) regions, thereby extending the epigenetic profiles beyond promoter regions.

To perform CUT & Tag analysis, we utilized the Hyperactive InSitu Universal CUT&Tag Assay Kit for Illumina Pro (Vazyme Biotech #TD904‐02). A total of 10 000 HSCs, BMDMs were collected as samples for the experimental group, with three replicates conducted. Subsequently, the prepared libraries underwent PCR amplification after extraction using phenol‐chloroform and ethanol precipitation. The PCR cycling conditions consisted of an initial step at 72 °C for 5 min, followed by denaturation at 98 °C for 30 s. This was succeeded by 14 cycles of denaturation at 98 °C for 10 s and annealing at 60 °C for 30 s. The amplification process was completed by performing a final extension step at a temperature of 72 °C for a duration of 1 min, followed by holding the samples at a temperature of 4 °C. For the purpose of post‐PCR cleanup, a volume of DNA clean beads equal to 1.5 times the volume of the DNA was utilized. In order to guarantee a thorough and precise sequencing analysis, all libraries that were prepared underwent sequencing on the Illumina Nova Seq 6000 platform, while strictly adhering to the manufacturer's guidelines.

### Bone Marrow Transplantation

To generate BM chimeras, 2 × 10^6^ BM cells from C57BL/6 (CD45.2^+^) mice were transplanted (via a single tail vein injection) into lethally (9.5 Gy) irradiated B6/SJL (CD45.1^+^) mice. Twelve weeks after BM transplantation, the recipient mice were subjected to treatments and/or analyses as described in the results and Figure legends. The percentage of different CD45.2^+^ cell populations was assessed at 12 weeks post‐transplantation in the BM of recipient mice.

### Immunoassays

The concentrations of human IL‐6, IL‐1β, and TNFα in serum were measured using human ELISA kits (Invitrogen) according to the manufacturer's instructions. For in vitro re‐stimulation of BMDMs with LPS, isolated and differentiated BMDMs were seeded into 24‐well plates and stimulated with 100 ng ml^−1^ LPS (InvivoGen) for 6 h. The supernatant was collected for measuring IL‐1β, IL‐6, and TNFα concentration using mouse ELISA kits (Invitrogen).

### RNA Interference

ADRB2 and negative control scrambled Silencer Select siRNAs were purchased from Thermo Fisher (combination of 3 siRNAs for each gene; Silencer select ADRB2 siRNA, 4 427 037, assay ID: s1625; Silencer select Scrambled siRNAs (Negative control), Cat#4 390 843, Thermo Fisher, Ambion). The Neon Electroporation System from Invitrogen was used to deliver siRNAs into BMDMs. Transfected cells were recovered in complete DMEM. The medium was replaced 3 h after electroporation. The cells were cultured for 48 h before further examination.

### cAMP and PKA Assay in BMDMs and THP‐1 Cells

Intracellular cAMP levels and PKA activity were measured using Kits from Abcam, as described before.^[^ ^17^ ^]^ Cells were seeded in 96‐well plates at 100 000 cells per well (100 µL). After 10 min of incubation at 37 °C, 10 µL of inhibitors or modulators were added as indicated and incubated at 37 °C for another 15 min. After that, 10 µL of test compounds (PAGln at 100 µM or ISO at 10 µm) were added for 5 min at 37 °C. The reaction was halted by adding lysis buffer and shaking for 10 min to lyse the cells. The lysates were immediately assayed by adding 40 µL to the assay plates, along with buffer controls and cAMP calibrators, and read for fluorescence on an ELX Ultra microplate reader (BioTek, USA) after covering with aluminum foil and resting at room temperature for 10 min.

For assays involving G‐protein modulation, cholera toxin (CTX; 1 µg mL^−1^) was pre‐incubated for 60 min by adding 10 µL (10X concentration) of modulators per well before the addition of the test compound to induce sustained cAMP production through the Gαs pathway, establishing a normalized baseline. SCH‐202676 (1 µM) was pre‐incubated for 45 min. The addition of agonists did not lead to a further detectable increase in cAMP, suggesting a masking of the Gαs‐mediated signaling. Assays with ADR inhibitors involved pre‐treating cells with ICI118551 (10 µM) or carvedilol (10 µM) for 15 min before adding the test compounds. Transfected cells were cultured for 48 h post‐transfection before the assays were conducted using the described protocol.

### 5‐Ethynyl‐2′‐Deoxyuridine (EdU) Proliferation Assay

HaCaTs and MDFs seeded in 24‐well plates were incubated under standard conditions and were divided into different groups. 24 h after incubation, cell proliferation was detected using the incorporation of 5‐ethynyl‐2‐deoxyuridine (EdU) with the EdU Cell Proliferation Assay Kit (Invitrogen, Click‐iT EdU Imaging Kits C10337). Steps were conducted according to the manufacturer's protocol. Briefly, the cells were incubated with 50 µm EdU for 2 h before fixation, permeabilization, and EdU staining. Then cell nuclei were stained with DAPI (Sigma‐Aldrich, D9542) at a concentration of 1 µg mL^−1^ for 8 min. The proportion of cells that incorporated EdU was determined by a Zeiss 710 laser‐scanning microscope (Zeiss, Thornwood, NY, USA).

### In Vitro Wound‐Healing Assay

HaCaTs were seeded in 6‐well plates to near confluence. Wounds were generated using a sterilized micropipette tip. Wound healing tests were performed in complete medium and photographed at 0 and 12 h. To quantify cell migration, the wound width from 10 randomly selected areas was measured at each time point, and the migration distance was the difference in width between 0 and 12 h. The migration distance of each sample was first normalized to the initial wound width and then compared with the other.

### Collagen Gel Contraction Assay

MDFs were seeded in 24‐well plates in 500 µL of collagen suspension (IBFB, Leipzig, Germany). After collagen gel polymerization, the gels were released immediately from the plates by tilting the plates slightly. The area of each collagen gel was measured at day 3. Statistical analysis was performed using ImageJ software.

### Mouse Routine Blood Test

Blood was collected from the retro‐orbital plexus under deep anesthesia (ketamine and xylazine) in EDTA‐coated tubes. Differential blood counts were measured within four h using an automated hematology analyzer (HEMAVET).

### Transient Transfection and Measurement of Reporter Gene Activity

To determine the transcriptional activity of NF‐κB, the cells were transfected with the plasmids containing the luciferase reporter gene controlled by the promoter with NF‐κB‐responsive elements (pGL4.32[*luc2P*/NF‐κB‐RE/Hygro], cat. E8491, pGL4.70[hRluc], cat. E6881, Promega, Madison, WI). The transfection was carried out at 37 °C using Lipo‐RNAiMAX Reagent (Invitrogen) following the manufacturer's instructions. All subsequent experiments were performed 48 h after the transfection. The luciferase activity was measured according to a standard protocol (Promega) on an ELX Ultra microplate reader (BioTek, USA). The luciferase activity was calculated in arbitrary units, evaluated as the ratio of the luciferase activity to the humanized Renilla luciferase (hRluc).

### Quantification and Statistical Analysis—Bioinformatic Analysis of CUT&Tag

Sequencing data were processed using Trimmomatic (v0.39) for quality control, aligned with Bowtie2 (v2.3.4.1), and peak calling was performed using MACS2 (v2.1.1). The resulting.mappedPeak files were analyzed and annotated with the ChIPseeker R package. For visualization,.bdg files were converted to.bw format using bedGraphToBigWig for compatibility with the UCSC Genome Browser. Final visualizations were performed using IGV (v2.8.2) with the sorted BAM or BigWig files.

### Bioinformatic Analysis of Bulk RNA‐seq

Read counts in each gene were quantified using the HTSeq‐count package. Differential gene expression analysis was performed with DESeq2, and significantly differentially expressed genes (DEGs) were identified based on an FDR‐adjusted *p* value < 0.05 in the PAGln‐treated group compared to controls. Significantly up‐ and down‐regulated DEGs were subjected to Gene Ontology (GO) enrichment and Kyoto Encyclopedia of Genes and Genomes (KEGG) analysis using PANTHER (http://www.pantherdb.org) with default background and significance thresholds. Enriched GO Biological Process terms were defined by FDR‐adjusted p‐value < 0.05. Volcano and bar plots were generated using customized R scripts.

### Pharmacokinetic Analysis

Pharmacokinetic parameters were calculated using standard Noncompartmental analysis.^[^ ^55^ ^]^ For humans, mean residence time (MRT) was determined as Volume of Distribution/CLG (Clearance for PAGln), and the elimination half‐life (t_1/2_) was calculated as (0.693 × Volume of Distribution)/CLG. For mice, plasma concentrations of PAGln/PAGly/PAA were measured at multiple time points following administration. C_max_ was defined as the highest observed concentration (µm) across all sampling points for each subject. *T*_max_ was recorded as the time point corresponding to the observed C_max_. The area under the plasma concentration–time curve (AUC) was calculated using the linear trapezoidal method. The terminal elimination half‐life (t_1/2_) was estimated using the slope of the log‐linear portion of the concentration‐time curve. Observed clearance (CL_obs_) was calculated as the Dose of administration/AUC. The area under the first moment curve (AUMC) was computed as the integral of concentration × time versus time, and MRT was determined as AUMC/AUC. All calculations were performed using GraphPad Prism version 8.0.

### Statistical Analysis

To examine the differences for pairwise comparisons, we used Student's *t*‐test (2‐tailed) or Wilcoxon signed‐rank test for continuous variables, and the Mann‐Whitney rank‐sum test for categorical variables and non‐normally distributed data. One‐way ANOVA, *t*‐test followed by Tukey multiple comparison post hoc, and Kruskal‐Wallis test were used for multiple comparisons. Categorical data are presented as n (%). Odd ratio (OR) for chronic wounds and corresponding 95% confidence intervals (CI) were estimated using both univariable (unadjusted) and multivariable (adjusted) logistic regression models. Adjustments were made for individual traditional wound risk factors, including age, sex, smoking, hypertension history, and hyperlipidemia history. Spearman correlation coefficients were generated to investigate the associations between inflammatory cytokines, plasma PAGln, and wound status. Heatmaps and scatter plots were used to visualize the correlations. All data are presented as mean ± SD or SEM, as indicated. Statistical tests used to compare conditions are indicated in the figure legends. GraphPad PRISM version 8.0 was used for the generation of graphs and statistics.

## Conflict of Interest

The authors declare no conflict of interest.

## Supporting information



Supporting Information

## Data Availability

The data that support the findings of this study are available on request from the corresponding author. The data are not publicly available due to privacy or ethical restrictions.
