# Supplementary material for: Type 2 Diabetes‐Associated Phenylacetylglutamine Induces Deleterious Inflammation Cycle in Myeloid Cells through β2 Adrenergic Receptors and Impedes Wound Healing
Source: Adv Sci (Weinh). 2025 Aug 14;12(42):e08205. doi: 10.1002/advs.202508205 (PMC12622421; doi:10.1002/advs.202508205)
Supplement: Supplementary file 1 — Supporting Information [file ADVS-12-e08205-s001.pdf]

Supporting Information

Phenylacetylglutamine Induces Deleterious Inflammation Cycle in Myeloid Cells Through β_2 Adrenergic Receptors and Impedes Wound Healing

Short running title: Phenylacetylglutamine Impedes healing via β_2-ADR

Lu Huang[1,2], Xinran Ye[1], Chiakang Ho[1], Ya Gao[1], Dongsheng Wen[1], Jiaming Sun[1], Yuxin Liu[1], Yangdan Liu[1], Guoyuan Wang[3], Yangbai Sun[4], Jinyan Zhang[5,], Yifan Zhang[1,]*, Qingfeng Li[1,]**

1. Department of Plastic and Reconstructive Surgery, Shanghai Ninth People's Hospital, Shanghai Jiao Tong University School of Medicine, Shanghai, China.
2. Department of Ophthalmology, Schepens Eye Research Institute of Massachusetts Eye and Ear, Harvard Medical School, Boston, Massachusetts, USA.
3. Luo Ping County People's Hospital, Qujing, Yunnan, China.
4. Department of Musculoskeletal Surgery, Fudan University Shanghai Cancer Center, Shanghai, China
5. Department of Cardiology, Zhongshan Hospital, Fudan University, Shanghai Institute of Cardiovascular Diseases, Shanghai, China.

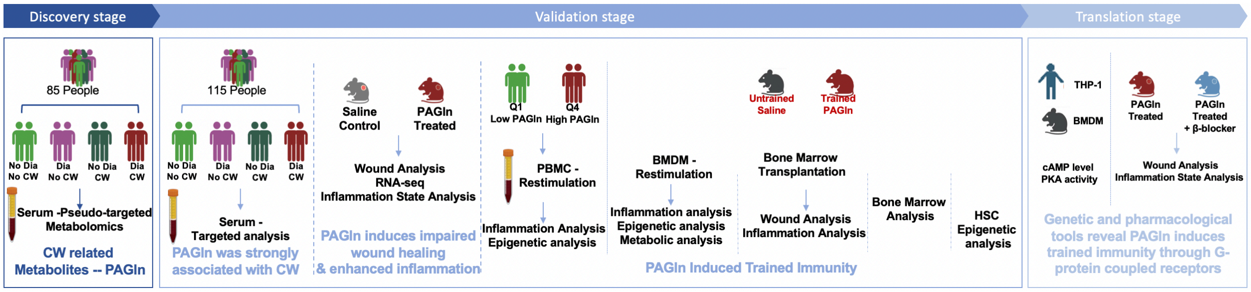

Figure S1 Study design

Study workflow includes three stages:

(1) Discovery: Identification of PAGln as a metabolite associated with chronic wounds using pseudo-targeted metabolomics in serum samples (discovery population, n = 85).

(2) Validation: Confirmation of the association between PAGln and chronic wounds via targeted LC/MS analysis in an independent cohort (validation population, n = 115). PAGln's causal effects on chronic wounds and inflammation were validated using mouse dermal wound models, human peripheral blood mononuclear cells (PBMCs), mouse bone marrow-derived macrophages (BMDMs), bone marrow transplantation, and hematopoietic stem cell (HSC) epigenetic analyses.

(3) Translation: Mechanistic investigation using genetic and pharmacological approaches demonstrated PAGln's role in trained immunity via G-protein coupled receptors, linking metabolic dysfunction to enhanced inflammation and impaired wound healing.

CW, chronic wounds; Dia, diabetic; PBMC, peripheral blood mononuclear cell; BMDM, bone marrow-derived macrophage; HSC, hematopoietic stem cell. The diagram was created in BioRender. https://www.biorender.com/.

Extended Data Figure 2

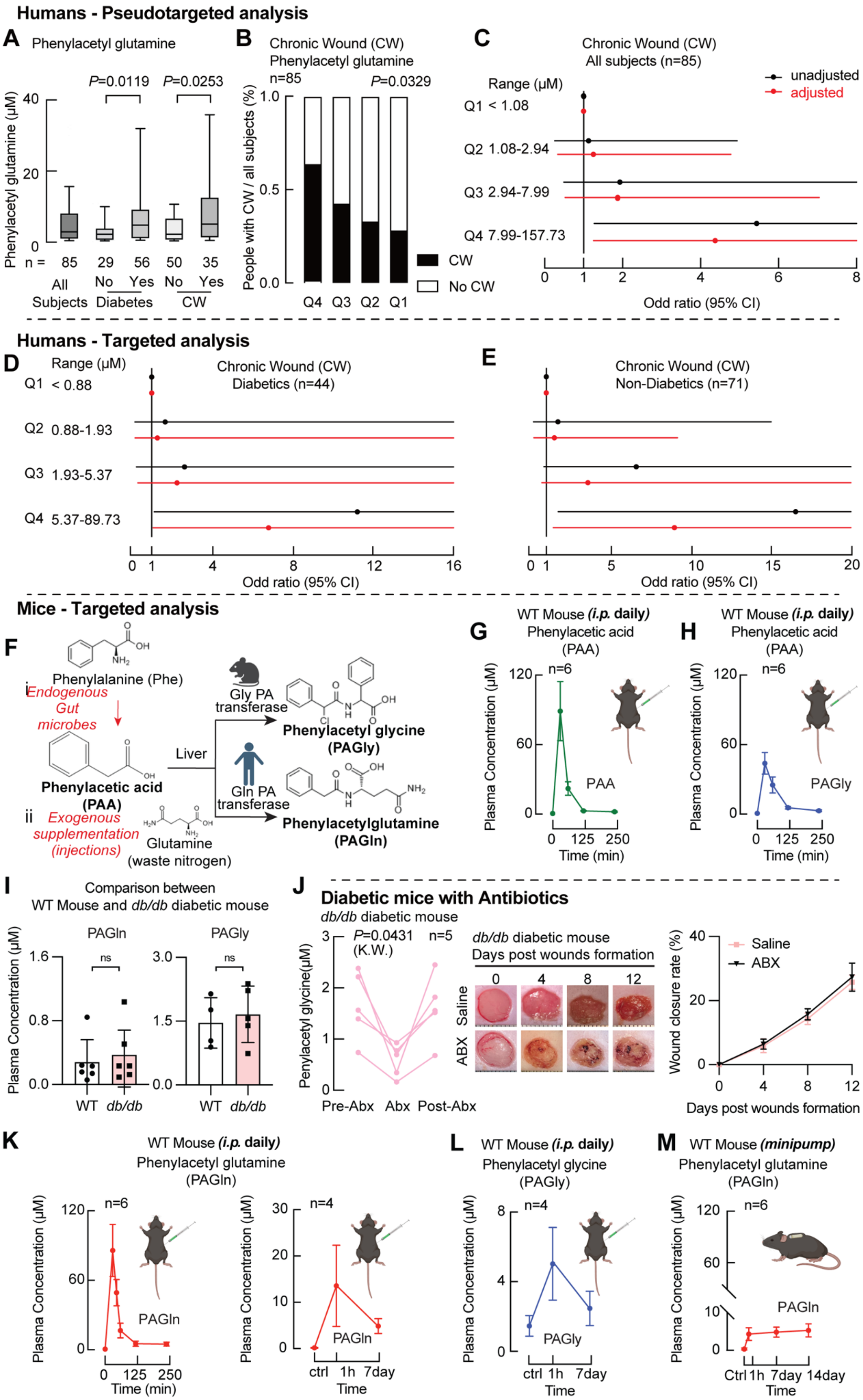

Figure S2 Elevated PAGln is associated with increased chronic wound risk in diabetic and non-diabetic individuals

(A) Plasma PAGln levels in discovery cohort subjects (n=85), grouped by diabetic status and chronic wound presence. Box plots show medians (horizontal lines), 25th–75th percentiles (box boundaries), and 10th–90th percentiles (whiskers). Statistical significance assessed by Mann–Whitney test.

(B) Stacked bar plot indicating chronic wound risk by PAGln quartiles (Q4 vs. Q1, log-rank test).

(C–E) Logistic regression analysis of chronic wound risk across PAGln quartiles: all subjects in discovery cohort (n=85; C), diabetic subjects in validation cohort (n=44; D), and non-diabetic subjects in validation cohort (n=71; E). Odds ratios (ORs) are unadjusted (black) or adjusted for age, sex, smoking, hypertension, and hyperlipidemia (red). Confidence intervals (CI, 2.5%–97.5%) indicated by line length.

(F) Scheme showing organismal production (i) and medical PAA supplementation-driven conversion (ii) of PAGln and PAGly in humans and mice.

(G–H) Plasma levels of PAA (G, n=6) and PAGly (H, n=6) after PAA intraperitoneal injection (50 mg/kg) over time.

(I) Comparison of plasma PAGln (left, n=6) and PAGly (right, n=6) levels between wild-type and *db/db* diabetic mice.

(J) *db/db* diabetic mice were treated with broad-spectrum antibiotics. Plasma PAGln levels (left, n=5) were measured at baseline (Pre-Abx), after 5-day antibiotics (Abx), and one week post-antibiotics (Post-Abx). Wound created after 5-day antibiotics (Abx), and wound closure rates (right, n=5) were measured. Two-way ANOVA for wound closure, Kruskal–Wallis test for multiple comparisons.

(K–L) Plasma levels of PAGln (left, 0-240 min, n=6, right, 0-7 day, n=5) and PAGly (L, 0-7 day, n=5) after intraperitoneal injection (50 mg/kg) over time.

(M) Plasma PAGln levels following continuous subcutaneous infusion via minipumps (0-14 day, 50 mg/kg, n=5). The diagram in panel F, G, H, K, L, and M was created in BioRender. https://www.biorender.com/.

Extended Data Figure 3

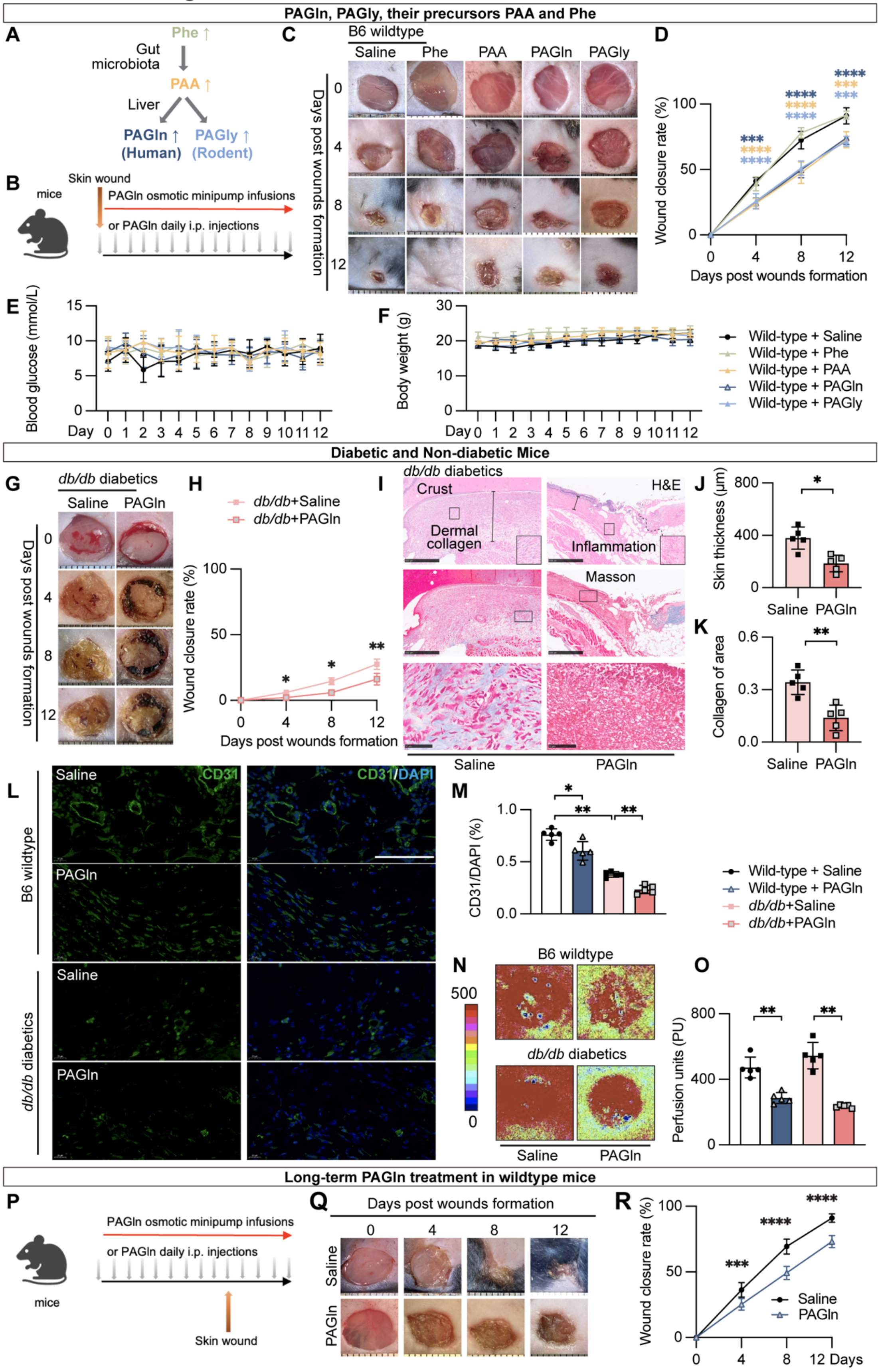

49

50 **Figure S3 PAGln delays wound healing in both diabetic and non-diabetic mice**

(A) Scheme illustrates the conversion of phenylalanine (Phe) to phenylacetic acid (PAA) at the organismal level, and the subsequent hepatic conversion of PAA to PAGln and PAGly in humans and mice.

(B) Dermal punch biopsy model scheme.

(C–F) PAGln, PAGly, and PAA subjection impaired wound healing in mice. (C-D) Representative images and quantification of wound closure (mean ± s.e.m., n=6 mice/group). (E) Blood glucose levels and (F) body weight in mice post-intraperitoneal (i.p.) injection of indicated compounds (mean ± s.e.m., n=5–10).

(G–O) PAGln delays wound healing in both diabetic and non-diabetic mice. Representative images and quantification for wound healing parameters in wild type and db/db diabetic mice: (G–H) Wound closure, (I–J) skin thickness, (I, K) dermal collagen (scale bars: top images 500 µm, bottom images 50 µm), (L–M) vascularization (scale bar: 100 µm), and (N–O) wound bed perfusion by laser Doppler imaging were assessed at day 8 post-injury (n=5 mice/group, mean ± s.e.m., n = 5 mice per condition, 3 independent experiments).

(P-R) Long-term PAGln treatment in wild-type mice. (P) Dermal punch biopsy was performed after 10 days of PAGln administration. (Q-R) Representative images and quantification of wound closure (mean ± s.e.m., n=6 mice/group).

Two-way ANOVA for wound closure, Mann–Whitney test for pairwise comparisons; *$P < 0.05$, **$P < 0.01$, ***$P < 0.001$, ****$P < 0.0001$. The diagram in panel B and P was created in BioRender. https://www.biorender.com/.

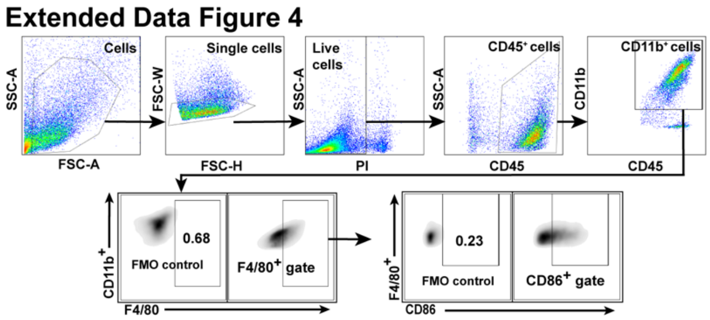

Figure S4 Representative FACS plots to identify CD86^{Hi} [Live, CD45^{+}, CD11b^{+}, F4/80^{+}] macrophage cells.

Extended Data Figure 5

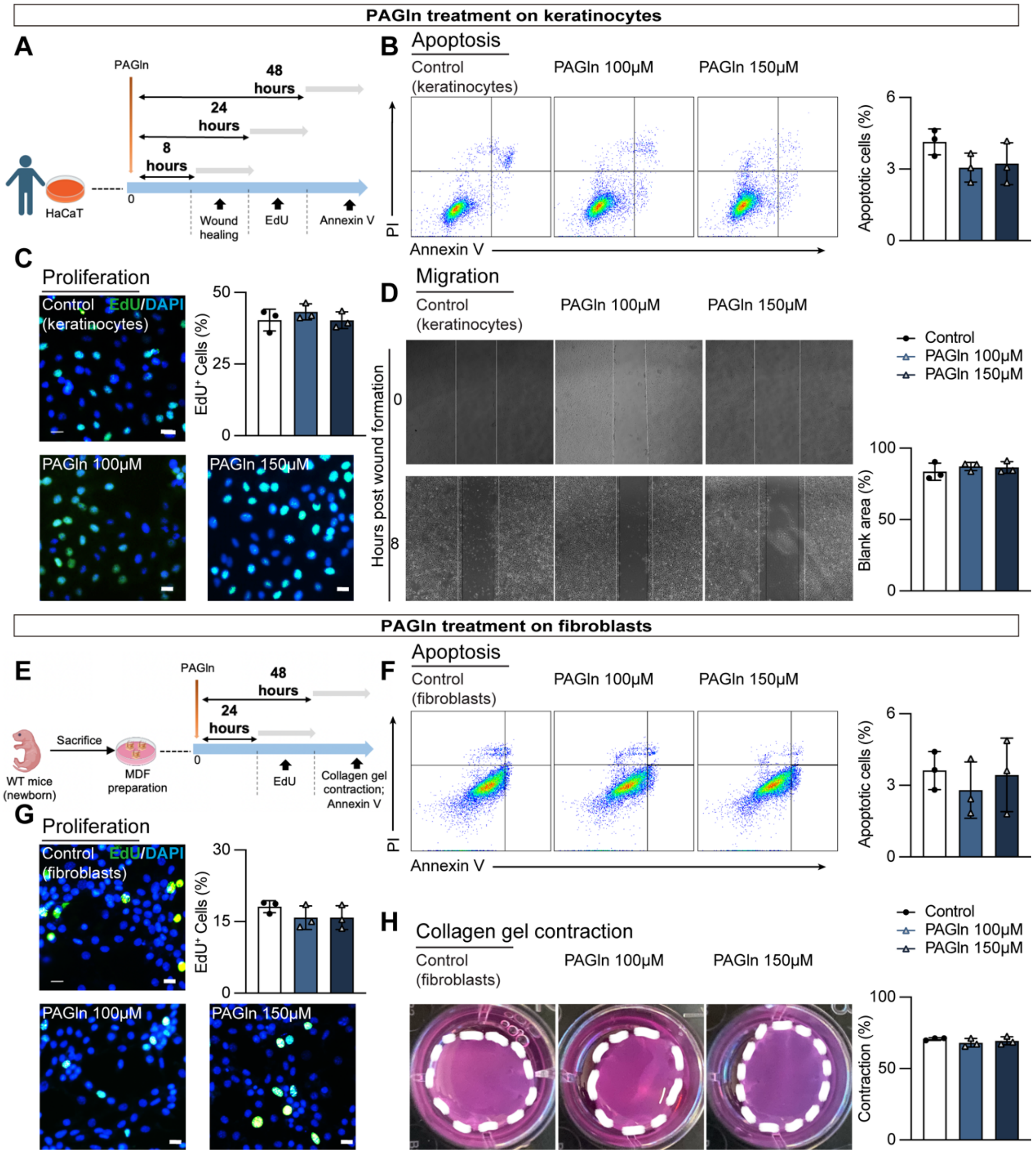

Figure S5 PAGln does not affect keratinocyte or fibroblast function

(A–D) In vitro effects of PAGln on human keratinocytes (HaCaT cell line). (A) Experimental schematic. Representative images and quantification of (B) apoptosis, (C) proliferation (EdU, green; DAPI, blue; scale bar: 50 μm), and (D) migration (scale bar: 20 μm).

(E–H) In vitro effects of PAGln on mouse dermal fibroblasts isolated from newborn mice. (E) Experimental schematic. Representative images and quantification of (F) apoptosis, (G) proliferation (EdU, green; DAPI, blue; scale bar: 50 μm), and (H) collagen gel contraction (scale bar: 20 μm). Mean ± s.e.m. of n=3 per condition; Kruskal–Wallis test for multiple comparisons. The diagram in panel A and E was created in BioRender. https://www.biorender.com/.

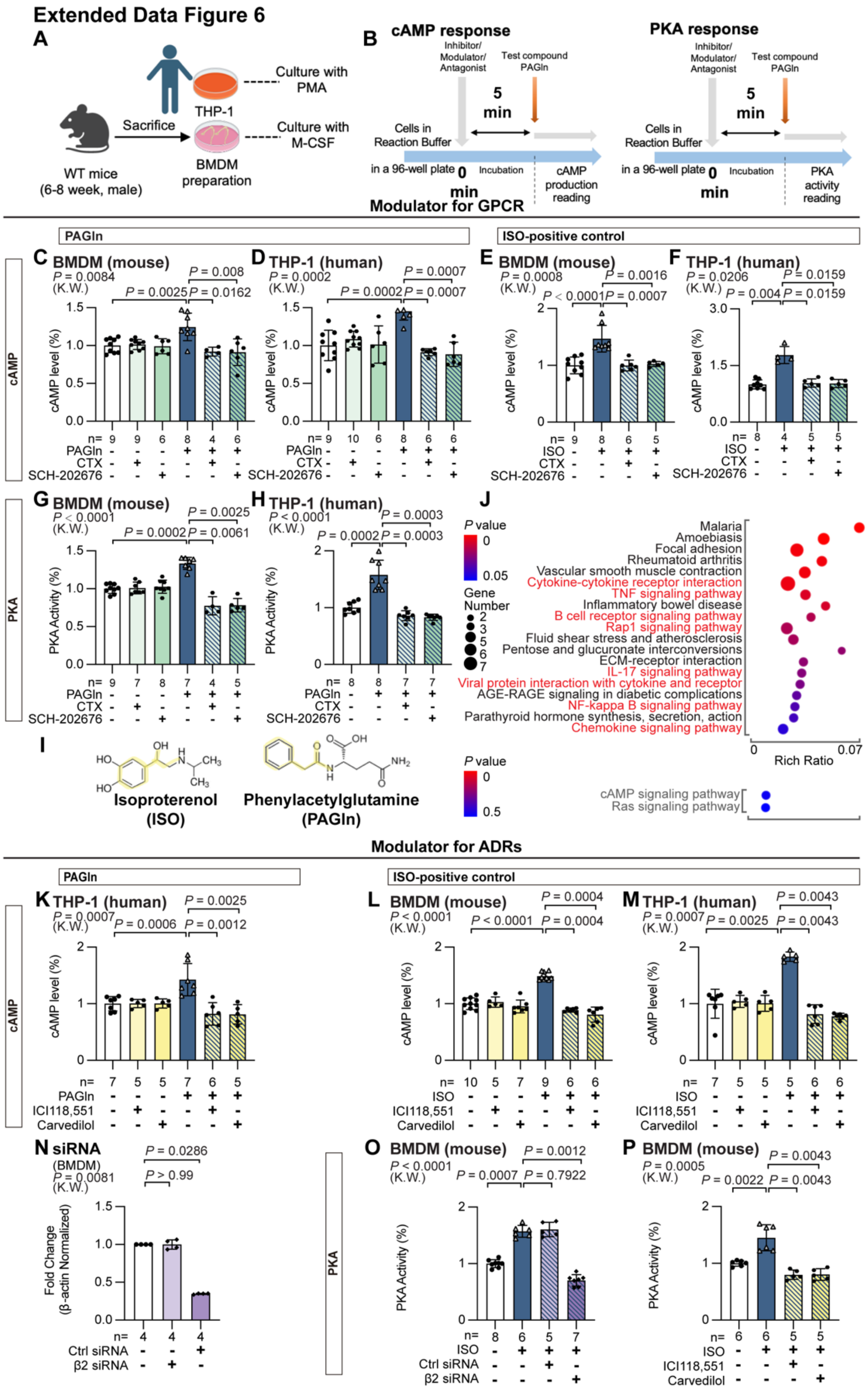

Figure S6 PAGln mediates trained immunity via β2-adrenergic receptors

(A) Experimental scheme for in vitro assays and (B) timelines for cAMP and PKA measurements. The diagram was created in BioRender. https://www.biorender.com/. (C–H) PAGln activates cellular signaling through G-protein-coupled receptors (GPCR). cAMP levels in BMDMs (C, E) and THP-1 cells (D, F) following PAGln (100 μM; C, D) or isoproterenol (ISO; positive control, 10 μM; E, F) treatments, in the presence of CTX (1 μg/mL) or SCH-202676 (1 μM). PKA activity measured in BMDMs (G) and

THP-1 cells (H) treated with PAGln (100 μM) and GPCR modulators. Data normalized to baseline immediately before adding PAGln or ISO.

(I) Structural similarity between PAGln, catecholamines (ISO), and PAGly.

(J) Top 19 KEGG pathway analysis of mouse skin post-PAGln or saline treatment (top) and cAMP and Ras pathway in mouse skin post-PAGln or saline treatment (bottom).

(K–O) PAGln mediates cellular responses via β2-adrenergic receptors.

(K-M)cAMP levels in THP-1 cells (K) and BMDMs (L, M) treated with PAGln (100 μM; K) or ISO (10 μM; L, M), with selective β2 antagonist ICI118,551 (10 μM) or nonselective β-blocker carvedilol (10 μM).

(N) ADR2B mRNA expression in BMDMs after transfection with β2-adrenergic receptor siRNA.

(O-P)PKA activity in BMDMs treated with ISO after transfection with control or β2-adrenergic receptor siRNA (O), or treated with β-blockers ICI118,551 and carvedilol (P). Data normalized to baseline immediately before ISO addition.

Mean ± s.e.m., n=4–10 mice per condition, 3 independent experiments; Mann–Whitney test for pairwise comparisons, Kruskal–Wallis test for multiple comparisons; *$P < 0.05$, **$P < 0.01$, ***$P < 0.001$, ****$P < 0.0001$.

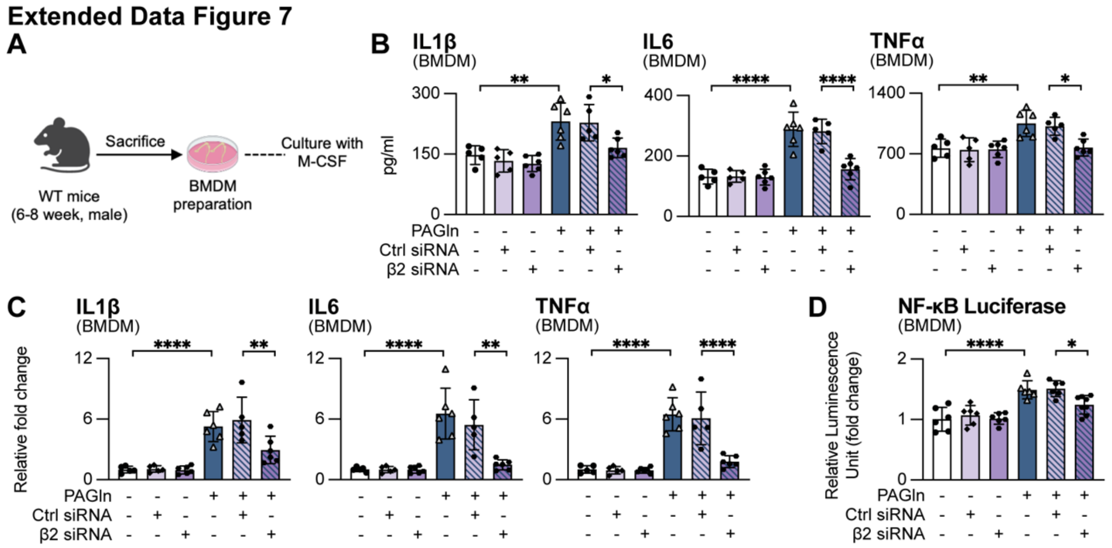

Figure S7 PAGln promotes the production of proinflammatory cytokines and activates NF-κB inflammatory signaling by macrophages through β2AR.

(A) Experimental scheme for in vitro assays. The diagram was created in BioRender. https://www.biorender.com/.

(B-C) Cytokine release (B) and expression (C) in BMDMs after transfection with β2-adrenergic receptor siRNA (mean ± s.e.m., n=6 per group).

(D) NF-κB reporter activity in BMDMs in response to PAGln after transfection with β2-adrenergic receptor siRNA (mean ± s.e.m., n=6 per group).

Mann–Whitney test for pairwise comparisons, Kruskal–Wallis test for multiple comparisons; $^*P < 0.05$, $^{**}P < 0.01$, $^{***}P < 0.001$, $^{****}P < 0.0001$.

Extended Data Figure 8

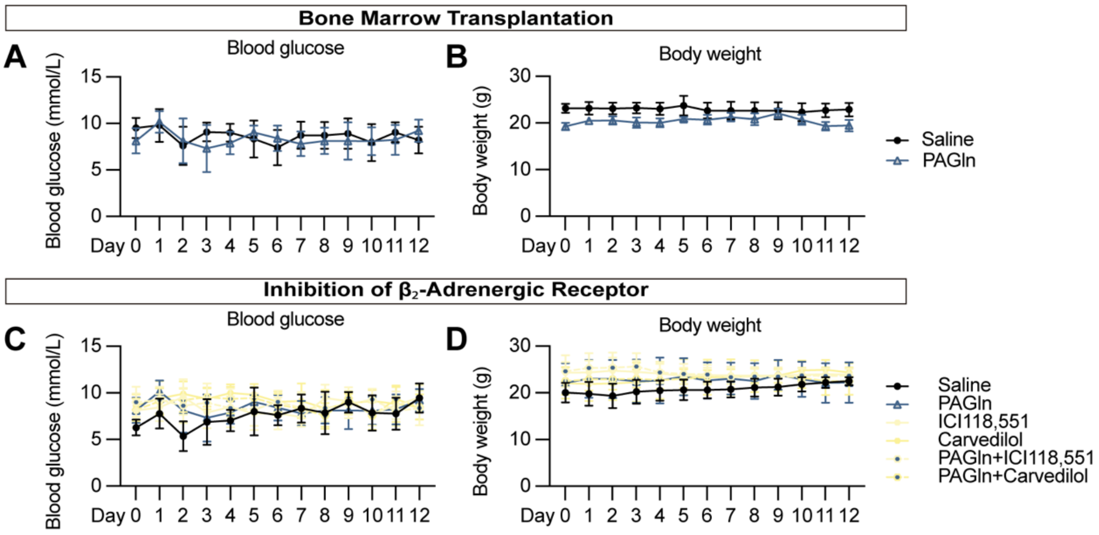

Figure S8 PAGln does not affect body weight or blood glucose in mice

(A–B) Blood glucose (A) and body weight (B) measurements in recipient mice transplanted with bone marrow from CD45.2+ donor mice treated with PAGln or saline (control). Measurements were taken at indicated time points post-wounding (mean ± s.e.m., n=5–10).

(C–D) Blood glucose (C) and body weight (D) in saline control or PAGln-trained mice treated with β-blockers (carvedilol, 50 mg/kg/day; ICI118,551, 10 mg/kg/day, intraperitoneally for 5 consecutive days). Data represent mean ± s.e.m., n=5.

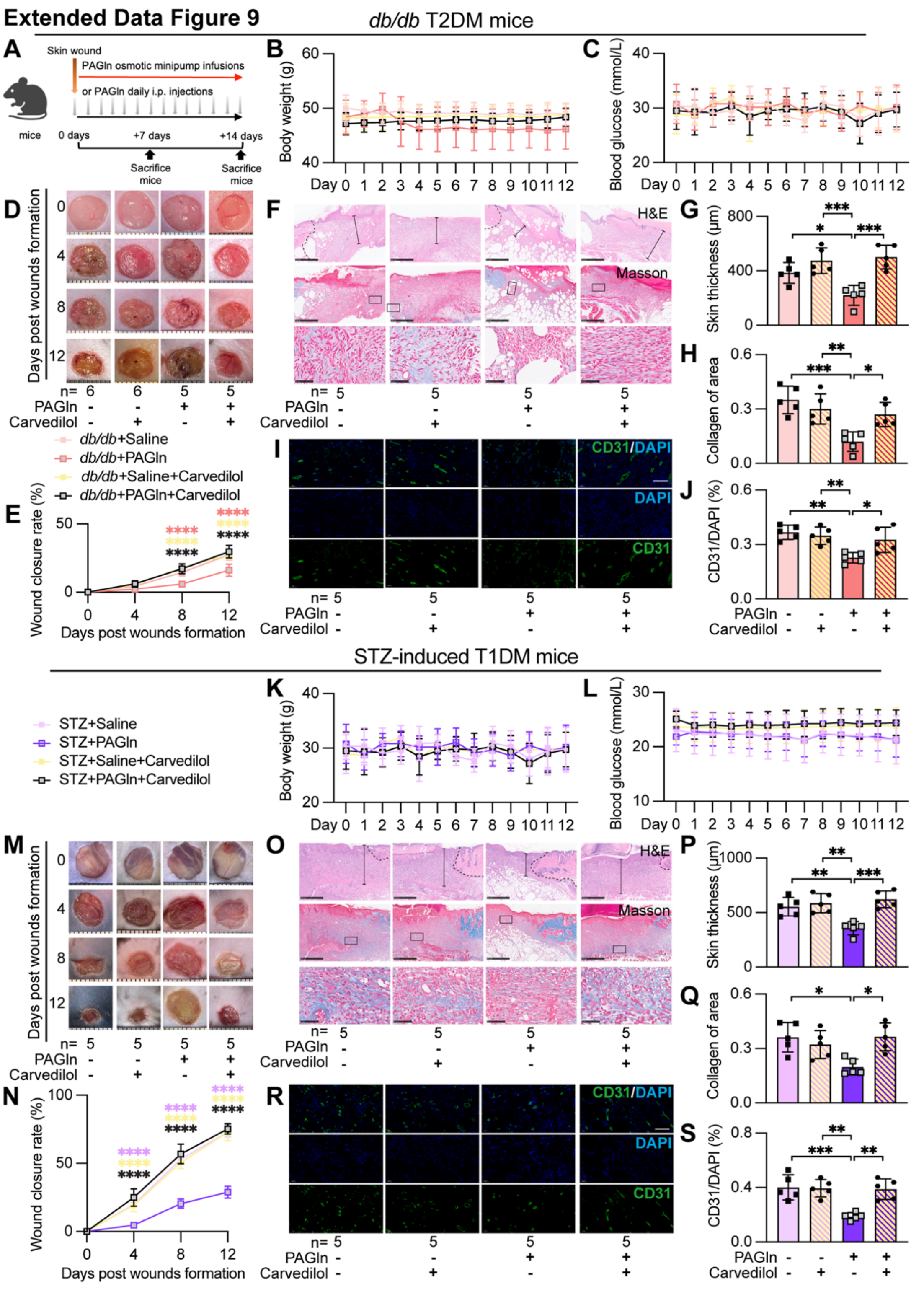

Figure S9 PAGln impairs wound healing via β-adrenergic receptors in diabetic mice

(A) Experimental schematic. The diagram was created in BioRender. https://www.biorender.com/.

(B–J) Effects of PAGln and β-blockers on wound healing in db/db type 2 diabetic (T2DM) mice. Representative images and quantification for (B) body weight, (C) blood glucose, (D, E) wound closure, (F, G, H) skin thickness and dermal collagen (scale bars:

500 μm top images; 50 μm bottom images), and (I, J) vascularization (scale bar: 20 μm), 7 days post-injury (mean ± s.e.m., n=3–6 mice/group, 3 independent experiments). (K–T) Effects of PAGln and carvedilol on wound healing in streptozotocin (STZ)-induced type 1 diabetic (T1DM) mice. Representative images and quantification for (K) body weight, (L) blood glucose, (M, N) wound closure, (O, P, Q) skin thickness and dermal collagen (scale bars: 500 μm top images; 50 μm bottom images), and (R, S) vascularization (scale bar: 20 μm), 7 days post-injury (mean ± s.e.m., n=3–5 mice/group, 3 independent experiments).

Two-way ANOVA for wound closure, Mann–Whitney test for pairwise comparisons, Kruskal–Wallis test for multiple comparisons; *$P < 0.05$, **$P < 0.01$, ***$P < 0.001$, ****$P < 0.0001$.

Extended Data Figure 10

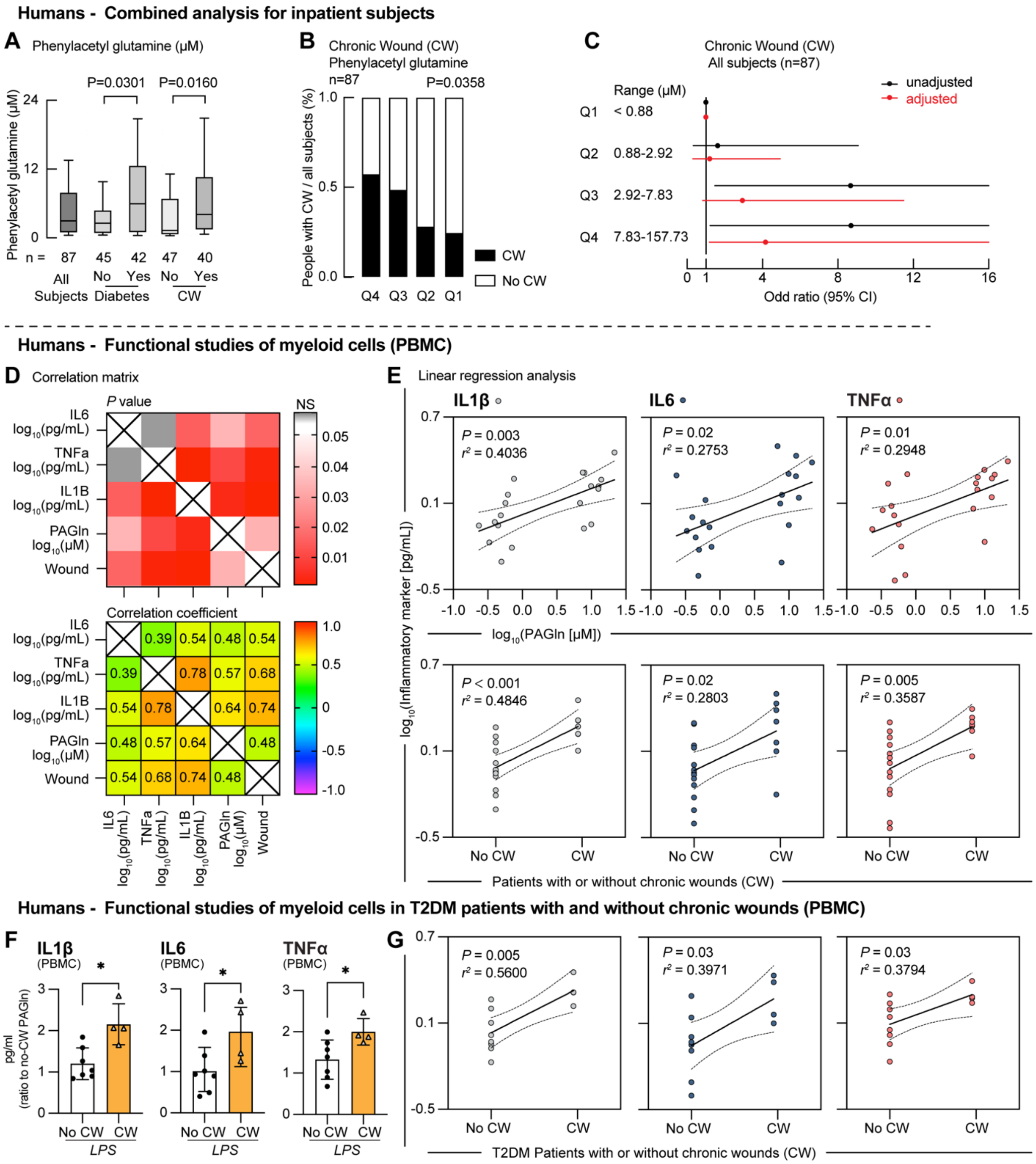

Figure S10. Elevated plasma PAGln correlates with higher chronic-wound risk and heightened pro-inflammatory responsiveness of PBMCs

(A) Relative plasma PAGln concentrations in the inpatient subjects (n = 87). Subjects were grouped based on diabetic status or the presence of chronic wounds. Box-and-whisker plots indicate medians (horizontal lines), 25th–75th percentiles (box boundaries), and 10th–90th percentiles (whiskers). Statistical significance was assessed by Mann–Whitney test.

(B) Stacked bar plot illustrating chronic wound risk across PAGln quartiles measured by stable isotope dilution LC-MS/MS (Q4 vs. Q1, log-rank test).
(C) Logistic regression analysis showing chronic wound risk by PAGln quartiles in inpatient subjects (n = 87). Unadjusted odds ratios (black); adjusted odds ratios controlling for age, sex, smoking, hypertension, and hyperlipidemia (red). Confidence intervals (CI, 5%–95%) are indicated by line length.
(D) Correlation matrix (n = 20 human subjects) linking inflammatory cytokines, plasma PAGln, and wound status. Upper: *P* values; lower: Spearman correlation coefficients.
(E) Linear regression of LPS-induced cytokine concentrations (IL-1β, grey; IL-6, blue; TNF, red) versus plasma PAGln and wound status (n = 20). Dotted lines denote the 95% confidence intervals.
(F) Cytokine secretion by human PBMCs isolated from T2DM subjects and restimulated *ex vivo* with LPS (100 ng/mL, 24 h, mean ± s.e.m., n=11), 3 independent experiments; Mann–Whitney test for pairwise comparisons.
(G) Linear regression of LPS-induced cytokine concentrations (IL-1β, grey; IL-6, blue; TNF, red) versus wound status in T2DM subjects (n=11). Dotted lines denote the 95% confidence intervals.

Table S1 Baseline characteristics of the discovery cohort

Characteristics	All	No Dia, No CW	Dia, No CW	No Dia, CW	Dia, CW
Number of participants	85	13	37	16	19
Age (years, mean ± SD)	60.0 ± 13.8	55.92 ± 11.99	60.05 ± 11.32	55.94 ± 19.2	59.75 ± 11.04
Sex male (%)	38.8	53.85	29.73	43.75	42.11
T2DM (%)	63.5	0	100	0	100
Body weight(kg, mean ± SD)	60.2 ± 11.6	62.9 ± 15.12	58.82 ± 9.46	61.91 ± 14.17	66.05 ± 12.66
Blood glucose level	7.76 ± 2.81	4.92 ± 0.59	9.01 ± 1.65	4.68 ± 0.58	9.88 ± 2.93
Wound size (the largest diameter, cm)	4.26 ± 2.23	/	/	4.94 ± 2.72	3.63 ± 1.3
Duration of wound presence (days, mean ± SD)	124.11 ± 37	/	/	121.38 ± 38.9	126.42 ± 36.23
History of hypertension (%)	34.1	30.77	24.32	43.75	47.37
History of hyperlipidemia (%)	12.8	15.38	8.11	6.25	26.32
Former/current smoking (%)	30.6	46.15	21.62	37.5	31.58
History of MACE (%)	9.4	0	5.41	6.25	26.32

Continuous variables are expressed as mean ± standard deviation; categorical variables are shown as percentages. T2DM, type 2 diabetes mellitus.

Table S2 Top plasma metabolites associated with chronic wounds in the discovery cohort (n = 85)

	Odd ratio Chronic wounds Q4 vs Q1		T2DM vs nonT2DM	Odd ratio Chronic wounds Q4 vs Q1 (Adjusted)	
Compound	OR	5-95% (CI)	p	OR	5-95% (CI)
Positively correlated with chronic wounds (unadjusted or adjusted) and T2DM					
**Phenylacetylglutamine**	**3.611**	**1.046 to 13.66**	**0.0119**	**4.136**	**1.015 to 19.94**
Positively correlated with chronic wounds (unadjusted) and T2DM					
Pipecolic acid	**4.375**	**1.258 to 16.85**	**0.0264**	4.03	0.9599 to 19.96
Correlated with T2DM					
Acetylcholine	0.333	0.08911 to 1.148	**0.0168**	0.266	0.03603 to 1.556
Trimethylamine-N-Oxide	0.917	0.2732 to 3.061	**0.0164**	1.636	0.3443 to 8.802
Lipoamide	1.091	0.3266 to 3.660	**0.0321**	0.846	0.1942 to 3.544
Correlated with chronic wounds					
Glutamine	**6.800**	**1.868 to 28.54**	0.6616	**10.730**	**2.152 to 87.14**
Creatinine	**5.600**	**1.562 to 22.92**	0.7929	**5.289**	**1.161 to 29.54**
Uric acid	**5.100**	**1.371 to 22.44**	0.2947	**4.789**	**1.030 to 28.80**
Negatively correlated with chronic wounds					
Urea	**0.2604**	**0.06534 to 0.9245**	0.8864	0.3253	0.06338 to 1.480

Unadjusted and adjusted odds ratios (OR) for chronic wounds comparing Q4 vs. Q1 levels of plasma metabolites are shown. Adjusted ORs account for age, sex, smoking, hypertension, and hyperlipidemia. Metabolites also exhibited significantly higher levels in diabetics compared to non-diabetics. CI, 95% confidence interval; OR, odds ratio; T2DM, type 2 diabetes mellitus.

Table S3 Baseline characteristics of the validation cohort

Characteristics	All	No Dia, No CW	Dia, No CW	No Dia, CW	Dia, CW
Number of participants	115	25	57	19	14
Age (years, mean ± SD)	58.7 ± 13.2	57.64 ± 15.24	58.23 ± 12.36	61.84 ± 12.44	58.64 ± 14.21
Sex male (%)	54.8	76	38.6	68.42	64.29
T2DM (%)	61.7	0	100	0	100
Body weight(kg, mean ± SD)	60.75 ± 12.76	60.33 ± 14.98	63.86 ± 11.87	55.97 ± 13.06	55.28 ± 7.79
Blood glucose level	7.97 ± 3.32	4.92 ± 0.90	9.84 ± 2.87	4.61 ± 0.48	10.38 ± 1.87
Wound size (the largest diameter, cm)	3.45 ± 2.41	/	/	3.68 ± 2.81	3 ± 2.32
Duration of wound presence (days, mean ± SD)	113.94 ± 31.1	/	/	114.05 ± 38.32	112.07 ± 20.8
History of hypertension (%)	20	8	24.56	15.79	28.57
History of hyperlipidemia (%)	10.4	12	15.79	0	0
Former/current smoking (%)	36.5	52	29.82	31.58	42.86
History of MACE (%)	8.7	12	8.93	5.26	7.14

Continuous variables are presented as mean ± standard deviation; categorical variables are shown as percentages. T2DM, type 2 diabetes mellitus.

Table S4. Distribution of plasma PAGln levels in the validation cohort.

	Phenylacetylglutamine (μM)						
PAGln range (%)	Whole subjects (n=115)	History of Chronic wounds in the whole subjects		History of Chronic wounds in non-T2DM		History of Chronic wounds in T2DM	
		No (n=82)	Yes (n=33)	No (n=25)	Yes (n=19)	No (n=57)	Yes (n=14)
Min	0.112	0.112	0.251	0.112	0.251	0.159	0.495
10	0.438	0.339	0.495	0.235	0.438	0.400	1.052
25	0.827	0.679	1.655	0.487	0.757	0.974	1.695
50	1.905	1.170	3.346	0.827	2.629	1.819	5.683
75	5.354	3.317	6.843	1.170	5.373	4.726	8.871
90	11.432	9.956	9.894	3.282	6.843	13.043	12.736
95	17.977	15.735	12.736	5.055	6.939	26.876	18.802
Max	89.732	89.732	21.615	15.735	11.432	89.732	21.615

Results in the Table show plasma PAGln concentrations at the indicated percentile cut off (in μM) within the whole cohort and the indicated subgroups. T2DM, type 2 diabetes mellitus.

Table S5 Pharmacokinetics of PAA in mice following 50 mg/kg PAA i.p. administration (*n* = 6)

Sample	C_{max} (μM)	T_{max} (min)	AUC (min μM)	$t_{1/2}$ (min)	$CL_{(obs)}$ (μM/min)	MRT (min)
Plasma	88.9 ± 25.8	30.0 ± 0.0	3965 ± 582.7	55.4±9.9	0.097 ± 0.028	80 ± 14

Table S6 Pharmacokinetics of PAGly in mice following 50 mg/kg PAA i.p. administration (*n* = 6)

Sample	C_{max} (μM)	T_{max} (min)	AUC (min μM)	$t_{1/2}$ (min)	$CL_{(obs)}$ (μM/min)	MRT (min)
Plasma	43.7 ± 9.2	30 ± 0	3004 ± 564	59.0 ± 10.0	0.109 ± 0.035	85.1 ± 14.4

Table S7 Pharmacokinetic analysis showed that murine PAGln clearance and MRT, and published human values.[1] MRT: Mean Residence Time; CLG: Clearance for PAGln.

**Sample**	C_{max} **(μM)**	T_{max} **(min)**	**AUC (min μM)**	**CLG(L/h)/Volume of Distribution (L)**	$t_{1/2}$ **(min)**	$CL_{(obs)}$ **(μM/min)**	**MRT (min)**
Plasma in mice following 50 mg/kg PAGln i.p. administration (n=6)	86.4 ± 22.9	30 ± 0	3946.6 ± 882.4	/	102 ± 34	0.063 ± 0.027	147 ± 49
Plasma in Human 0-2 years old (n=3)— Monteleone et. al.	/	/	/	3.0/6.1	84.3	/	122
Plasma in Human 3-5 years old (n=10)— Monteleone et. al.	/	/	/	5.0/8.7	72.1	/	104.4
Plasma in Human 6-11 years old (n=7)— Monteleone et. al.	/	/	/	7.7/18.7	101.1	/	145.8
Plasma in Human 12-17 years old (n=4)— Monteleone et. al.	/	/	/	12.7/20.9	68.3	/	98.7
Plasma in Human adults (n=53)— Monteleone et. al.	/	/	/	11.9/24.6	85.9	/	124

1. Monteleone, J. P. R., Mokhtarani, M., Diaz, G. A., Rhead, W., Lichter-Konecki, U., Berry, S. A., LeMons, C., Dickinson, K., Coakley, D., Lee, B., and Scharschmidt, B. F. (2013) Population Pharmacokinetic Modeling and Dosing Simulations of Nitrogen-Scavenging Compounds: Disposition of Glycerol Phenylbutyrate and Sodium Phenylbutyrate in Adult and Pediatric Patients with Urea Cycle Disorders. *The Journal of Clinical Pharmacology* **53**, 699-710
